# Whole-body vibration training and bone mineral density in older adults: an updated systematic review and meta-analysis

**DOI:** 10.1186/s12891-026-09504-7

**Published:** 2026-01-21

**Authors:** Wendi Chen, Xingyi Li, Changzhou Chen

**Affiliations:** 1 School of Physical Education, Shanghai University of Sport, Shanghai, China; 2 Department of Sport and Health, Shinhan University, Uijeongbu-si, Gyeonggi Province Republic of Korea

**Keywords:** Whole-body vibration training, Elderly, Bone mineral density, Meta-analysis

## Abstract

**Objectives:**

To evaluate the effects of whole-body vibration training on bone mineral density at different sites in the elderly population via meta-analysis.

**Methods:**

We systematically searched CNKI, VIP, Wanfang, China Biomedical Literature Database, PubMed, Cochrane Library, Embase, and Web of Science from inception to April 20, 2025, for randomized controlled trials comparing whole-body vibration training with conventional exercise on bone mineral density in elderly individuals. Methodological quality was assessed using the TESTEX (Tool for the Assessment of Study Quality and Reporting in Exercise) checklist, while risk of bias was evaluated with the Cochrane RoB 2.0 tool. Data were analyzed using Review Manager 5.4 for meta-analysis and heterogeneity assessment, Stata MP 18 for Egger’s test, and the GRADE framework for evidence quality evaluation.

**Results:**

Fourteen studies involving 1,447 participants were included, with TESTEX = 9.5 (good quality) and risk of bias (21% low risk, and 79% some concerns). Results indicated that whole-body vibration training significantly increased bone mineral density in the Ward's triangle region (WMD=0.04, P < 0.00001) and greater trochanter region (WMD = 0.03, P = 0.0002) in the elderly. It also showed improvement effects on the femoral neck region (WMD = 0.01, P = 0.01) and lumbar spine L2‒L4 region (WMD = 0.04, P = 0.003). However, no significant effects were observed in the lumbar spine L1‒L4 region (WMD=0.00, P=0.79) or total hip region (WMD=0.01, P=0.56).

**Conclusions:**

Whole-body vibration training significantly increased bone mineral density in Ward's triangle and greater trochanter regions in elderly subjects, with smaller improvements in the femoral neck region. The L2‒L4 lumbar spine segment may substantially increase, whereas the L1‒L4 lumbar spine segment and total hip region have negligible effect.

**Trial registration:**

CRD420251038478.

**Supplementary Information:**

The online version contains supplementary material available at 10.1186/s12891-026-09504-7.

## Introduction

The global population structure is accelerating its shift toward aging, leading to a significant increase in age-related chronic diseases. Osteoporosis has emerged as a particularly urgent public health issue requiring immediate attention [1]. Current epidemiological surveillance data indicate that osteoporosis and osteopenia affect 19.7% and 40.4% of the global population, respectively, with disease burden metrics showing a persistent increasing trend [2].

This metabolic bone disorder primarily manifests as fragility fractures, which predominantly occur in weight-bearing skeletal structures, including vertebrae and the proximal femur, and has high morbidity and mortality rates [3]. The International Osteoporosis Foundation reported a one-year mortality rate of 20–24% among hip fracture patients, while survivors often experience severe functional decline: 40% become immobile, 60% require assisted living within 12 months, and 80% demonstrate impairment in instrumental activities of daily living (IADLs) [4]. These chronic disabilities impose multifaceted burdens on affected individuals and their caregivers, including physical deterioration, psychological distress, and socioeconomic pressures.

Consequently, implementing evidence-based interventions to help older adults reduce the risks of osteoporosis and improve bone health is a key component of comprehensive geriatric care strategies for the elderly. Extensive research indicates that physical exercise plays a positive role in promoting bone growth and development as well as maintaining bone mass. For example, systematic resistance training effectively prevents and improves age-related osteoporosis [5, 6]; moderate walking and group-based aerobic exercise significantly increase bone mass [7, 8].

However, factors such as joint degeneration and diminished muscle strength make it challenging for older adults to sustain high-intensity exercise [9, 10]. Whole-body vibration training (WBVT), which is controllable, safe, and effective, serves as a low-impact alternative intervention for preventing fractures and osteoporosis in elderly individuals [11].WBVT is an intervention that promotes bone formation by allowing subjects to stand on a vibrating platform and receive external mechanical oscillation stimuli [12]. This training enhances bone density through mechanical vibration signals [13, 14] and can achieve targeted treatment for specific body regions by adjusting the subject’s posture [15].

However, the effects of WBVT on BMD in elderly individuals remain controversial. Multiple studies suggest that WBVT may not consistently yield beneficial effects on bone health in elderly individuals [16, 17]. While several investigations have indicated that WBVT can slow the decrease in BMD in older women, the optimal intervention parameters remain unclear [18, 19]. Given the numerous controversies and unresolved issues in existing research, scholars propose that current studies should compare the effects of different WBVT methods on skeletal health. This includes evaluating vibration duration, frequency, rest intervals, and auxiliary exercise types, as well as determining optimal dose‒response relationships and variations in vibration characteristics [20].

Therefore, this study employs systematic review and meta-analysis methods to investigate the intervention effects of WBVT on BMD at different sites in the elderly population. Through subgroup analysis, we examine differences in intervention effects across four dimensions: site-specific responsiveness, vibration parameters, training cycles, and movement patterns. This study aims to provide high-quality evidence-based guidance for developing personalized WBVT protocols for elderly individuals, thereby laying the foundation for precision strategies in healthy aging.

## Methods

This systematic review and meta-analysis adhered to the recommendations outlined in the Cochrane Handbook for Systematic Reviews of Interventions (Version 6.5) [21]and was reported strictly according to the PRISMA reporting guidelines (PRISMA 2020 Statement) [22]. It was registered in the International Prospective Systematic Reviews Registry in April 2024 (registration number CRD420251038478). After completing the literature screening process, we updated the registration record status to “Completed” in accordance with platform specifications, without making any modifications to the original research protocol.

### Search strategy

Two authors (CWD, LXY) independently screened all records. Searches were conducted in the China National Knowledge Infrastructure (CNKI), VIP Chinese Journal Database, Wanfang Database, China Biomedical Literature Database, PubMed, Cochrane Library, Embase, and Web of Science. The search period spanned from the inception date of each database to April 20, 2025, with no language restrictions. The search strategy was designed on the basis of the Population, Intervention, Outcome (PIO) framework. It combines MeSH terms (elderly, vibration, and bone density) with free-text keywords, employing Boolean search terms"OR” and “AND” to construct the search query. For the complete retrieval strategy, please refer to Supplement 1 Search strategy.

Two independent researchers (CWD and LXY) reviewed titles and abstracts to exclude clearly irrelevant studies and rigorously screened full texts against inclusion and exclusion criteria to determine eligibility. Disagreements were resolved through discussion; when necessary, a third assessor (CCL) was consulted to reach a consensus before study inclusion.

### Inclusion and exclusion criteria

Inclusion criteria: (1) randomized controlled trial design; (2) participants aged ≥ 60 years with good general and mental health who were capable of independent walking; (3) the intervention group received whole-body vibration training, whereas the control group underwent conventional exercise intervention or placebo control; and (4) bone mineral density values were used as outcome measures.

Exclusion criteria: (1) participants with cardiovascular disease or other high-risk conditions; (2) participants with diabetes, rheumatoid arthritis, or other diseases affecting bone metabolism; (3) participants taking medications for osteoporosis or other bone metabolism-affecting conditions during the study period that significantly altered outcomes; (4) duplicate publications, studies lacking full-text access or data, or studies with unclear baseline measurements.

### Data extraction

Designed data extraction forms specified key content: ① study details (first author, publication year); ② subject characteristics; ③ sample size; ④ intervention protocol; ⑤ control measures; and ⑥ outcome measures (BMD values at the femoral neck, Ward’s triangle, greater trochanter, total hip, lumbar spine L1‒L4, and lumbar spine L2‒L4). All the data were independently extracted by two researchers(CWD and LXY); discrepancies were resolved by a third researcher(CCL).

For non-English literature, we employed neural machine translation tools (DeepL) to assist in comprehending the full text content. All key data extraction was based on the original language texts and verified by two independent researchers (CWD and LXY). Where uncertainties arose, we consulted a third researcher or the original author to ensure the accuracy of data extraction.

Pooled data are expressed as the mean ± standard deviation (mean ± SD). The Evidence-Based Medicine Assistant indicator calculation and conversion tool was used to compute the mean differences and their standard deviations before and after the intervention.

The mean difference formula is as follows: M_change_== M_final−_ M_baseline_, where M_change_represents the mean difference between pre- and post-intervention; M_final_represents the post-intervention mean; M_baseline_represents the pre-intervention mean. The standard deviation change formula is: SD_change_== $\:\sqrt{{\mathrm{S}\mathrm{D}}_{\mathrm{b}\mathrm{a}\mathrm{s}\mathrm{e}\mathrm{l}\mathrm{i}\mathrm{n}\mathrm{e}}^{2}+{\mathrm{S}\mathrm{D}}_{\mathrm{f}\mathrm{i}\mathrm{n}\mathrm{a}\mathrm{l}}^{2}-\left(2\times\:\mathrm{C}\mathrm{o}\mathrm{r}\mathrm{r}\times\:{\mathrm{S}\mathrm{D}}_{\mathrm{b}\mathrm{a}\mathrm{s}\mathrm{e}\mathrm{l}\mathrm{i}\mathrm{n}\mathrm{e}}\times\:{\mathrm{S}\mathrm{D}}_{\mathrm{f}\mathrm{i}\mathrm{n}\mathrm{a}\mathrm{l}}\right)}$, where SD_change_is the difference in standard deviation between pre- and post-intervention;$\:{\mathrm{S}\mathrm{D}}_{\mathrm{b}\mathrm{a}\mathrm{s}\mathrm{e}\mathrm{l}\mathrm{i}\mathrm{n}\mathrm{e}}$ is the pre-intervention standard deviation, and $\:{\mathrm{S}\mathrm{D}}_{\mathrm{f}\mathrm{i}\mathrm{n}\mathrm{a}\mathrm{l}}$ is the post-intervention standard deviation. Corr denotes the correlation coefficient, which is set at 0.5 [23, 24].

For standard deviations requiring conversion, the Cochrane Standard Deviation Conversion Tool is used to estimate values on the basis of confidence intervals and sample sizes. When the sample size is ≤ 60, calculations use the t-distribution with the conversion formula:$\:\mathrm{S}\mathrm{D}$=$\:\frac{\sqrt{\mathrm{N}}\times\:\left({\mathrm{C}\mathrm{I}}_{\mathrm{u}\mathrm{p}\mathrm{p}\mathrm{e}\mathrm{r}}-{\mathrm{C}\mathrm{I}}_{\mathrm{l}\mathrm{o}\mathrm{w}\mathrm{e}\mathrm{r}}\right)}{2\times\:{\mathrm{t}}_{{\upalpha\:}/2,\mathrm{d}\mathrm{f}}}$, where α = 0.05 corresponds to a 95% CI and df = *N*−1. When the sample size is> 60, the formula simplifies to$\:\mathrm{S}\mathrm{D}$=$\:\frac{\sqrt{\mathrm{N}}\times\:\left({\mathrm{C}\mathrm{I}}_{\mathrm{u}\mathrm{p}\mathrm{p}\mathrm{e}\mathrm{r}}-{\mathrm{C}\mathrm{I}}_{\mathrm{l}\mathrm{o}\mathrm{w}\mathrm{e}\mathrm{r}}\right)}{3.92}$.

### Research methodology quality assessment

Two independent researchers (CWD and LXY) assessed the methodological quality of each study via the Tool for Evaluating Study Quality and Reporting in Motion (TESTEX) checklist. This tool comprises a study quality section (items 1–5) and a study report section (items 6–12), with items 6 and 8 featuring three and two subcriteria, respectively, yielding a maximum score of 15 points. Each criterion received 1 point; otherwise, 0 points were assigned. Studies were categorized into four groups on the basis of total scores: “Excellent Quality” (12–15 points), “Good Quality” (9–11 points), “Moderate Quality” (6–8 points), and “Poor Quality” (< 6 points) [25, 26].

### Research bias risk assessment

Two independent researchers (CWD and LXY) assessed the risk of bias in the included studies using the Cochrane Risk of Bias 2.0 (RoB 2) tool, scoring across five domains: risk of bias to allocation, risk of bias to treatment, risk of bias to outcome data, risk of bias to outcome measurement, and risk of bias to selective reporting. Each domain had five response options: Yes, Probably Yes, Probably No, No, and No Information. The risk of bias for each domain was categorized into three levels: “Low Risk,” “Some Risk,” and “High Risk.” The overall risk of bias was determined by combining the risk assessments across domains.

A study was classified as having a low risk of bias if it received a “low risk” rating in all domains; if at least one domain was rated “some concern,” the study was considered to have some bias issues; if at least one domain was rated “high risk” or if multiple domains were rated “some concern,” the study was deemed to have a high risk of bias, which may compromise the validity of the results. This study excluded high-risk studies [27].

### Assessment of evidence quality

Two independent researchers (CWD and LXY) systematically assessed evidence quality via GRADEprofiler software and conducted a comprehensive evaluation on the basis of core dimensions, including study design, risk of bias, inconsistency, and indirectness.

The evidence quality was categorized into four grades according to the GRADE criteria: high quality indicates a high degree of confidence that the estimated value is close to the true effect value; moderate quality indicates some confidence in the effect estimate, but the true effect may differ substantially; low quality indicates limited confidence in the effect estimate, with the true effect potentially differing significantly; and very low quality reflects very low certainty about the effect estimate. In accordance with the Cochrane Handbook guidelines, evidence quality grades were integrated with effect size results for analysis [28].

### Statistical methods

Analysis was performed via Review Manager 5.4 software. Effect sizes were determined using weighted mean differences (WMDs) or standardized mean differences (SMDs) with 95% confidence intervals (95% CIs). WMD was selected as the effect measure when all studies employed identical measurement methods and units; SMD was used when methods differed [29]. BMD values at six sites—the femoral neck, total hip, lumbar spine L1‒L4, lumbar spine L2‒L4, greater trochanter, and Ward’s triangle—were used as outcome effect measures.

In accordance with Sect. 23.3.4 and 23.3.5 of the Cochrane Handbook for Systematic Reviews of Interventions (Version 6.5) [30], this study employed either splitting the control group or selective inclusion for multi-arm trial data. Splitting the control group refers to evenly distributing the sample size of the shared control group among the relevant intervention groups while preserving the original mean and standard deviation.

Random-effects models were applied when moderate to substantial heterogeneity was detected, while fixed-effects models were used when heterogeneity was mild or negligible. Simultaneously, we conducted sensitivity analyses using fixed-effects and random-effects models. Consistent results indicate the robustness of the pooled estimates. The combined results are presented in a forest plot.

Heterogeneity among the included studies was assessed via I² and the Cochran Q test. The Cochran Q test criteria were as follows: *P* < 0.10 indicates heterogeneity; *P* > 0.10 indicates no heterogeneity. I²= 0% indicates no heterogeneity; 0% < I²< 25% indicates low heterogeneity; 25% < I² < 50% indicates moderate heterogeneity; 50% < I² < 75% indicates high heterogeneity; and I²≥ 75% indicates extremely high heterogeneity. When heterogeneity reached moderate levels or higher, the pooling model was modified, or the exclusion method was applied to identify sources of heterogeneity.

Egger’s test was performed via Stata MP 18 software to assess publication bias. When *p* > 0.05, no bias exists; when *p* ≤ 0.05, publication bias may exist. Further analysis by trimming in StataIC 15 was used to assess the impact of publication bias on the results. Trimming yielded non-significant differences, indicating robust results; reversal of the results would suggest publication bias.

We plan to conduct subgroup analyses for the following factors: (1) vibration frequency, (2) vibration amplitude, (3) training cycle, and (4) exercise mode. It is important to emphasize that all subgroup analyses are considered exploratory. Given the limited number of studies within each subgroup, these findings should be interpreted with caution, and the conclusions from these analyses require further validation in future research.

## Results

### Study selection

**Fig. 1:**
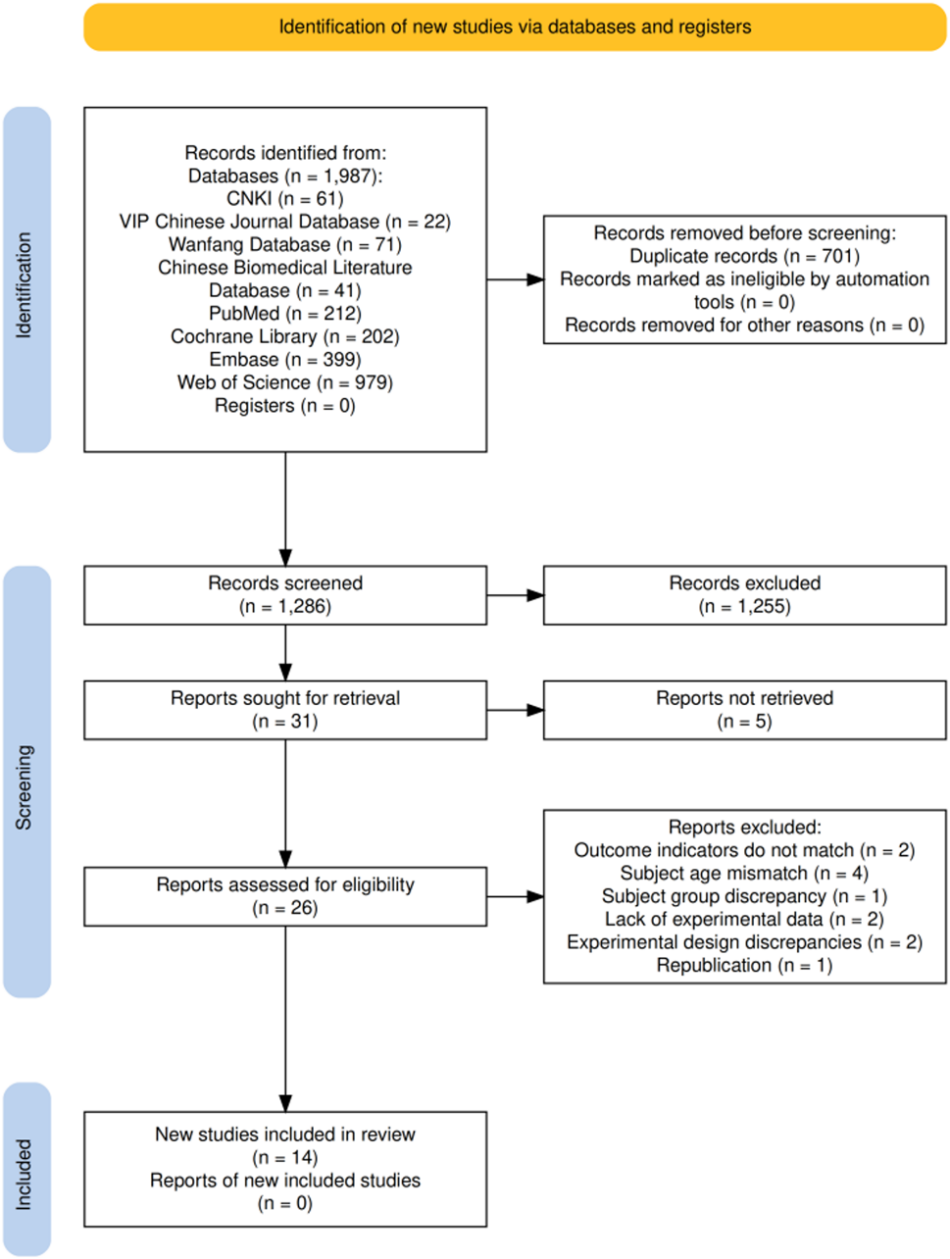
Literature screening flowchart

A total of 1,987 publications were retrieved through the search strategy. Ultimately, 14 studies were included. The screening process is illustrated in Fig. 1.

### Study characteristics

This study included 14 studies involving 1,447 participants aged 60–93 years (4% male and 96% female). The number of participants per study ranged from 28 to 710. The training protocol variables included interventions lasting 10–60 min, comprising static and dynamic movements such as squats and standing postures. The frequency ranged from 12.6 to 45 Hz, the amplitude ranged from 0.5 to 12 mm, and the weekly frequency ranged from 2 to 5 sessions. The protocol duration spanned 8 to 72 weeks. The characteristics of the included studies are summarized in Table 1.

**Table 1:** Characteristics of the included studies

Incorporated into the study	Feature	Number of cases (T/C)	Intervention method		Frequency (Hz) Amplitude (mm)	Duration	Frequency (times/week)	Bone sites
			T	C				
Ba&Cheng (2016)^[31]^	Female, 60–65 years	26/26	Knee squats, heel raises, squats	keep strokes	20–35 Hz 3 mm	24 W	3	②④⑤⑥
Ba&Cheng (2017)^[32]^	Female, 60–70 years	30/31	Half squats, deep squats, calf raises, and single-leg squats	keep strokes	20–35 Hz 3 mm	24 W	3	②⑤⑥
Cheng et al. (2021)^[33]^	Female, 60–70 years	Mid frequency 19 High frequency 18/19	Half squat, full squat, weight lifting, and single-leg lift	keep strokes	20 Hz/40 Hz 3 mm	24 W	3	②⑤⑥
Gómez-Cabello et al. (2014)^[34]^	People aged 65 and over	24/25	Stand upright with your knees slightly bent and hold the machine’s handle with both hands	No intervention	40 Hz 2 mm	11 W	3	①②③
Gusi et al. (2006)^[35]^	Female ≥ 60 years of age	14/14	Stand upright	Five-minute walk and five-minute stretch	12.6 Hz 3 mm	32 W	3	②⑤⑥
Leung et al. (2014)^[36]^	> 60 years of age, female	280/316	Stand upright	No intervention	35 Hz 0.3 g	36 W	5	①
Lu (2016)^[37]^	Female, 60–70 years	Low frequency 16 Medium frequency 17 High frequency 16/16	Half squat, deep squat, calf raise, single leg squat	keep strokes	10–15 Hz/ 25–30 Hz/ 40–45 Hz 3 mm	24 W	3	②⑤⑥
Lu et al. (2012)^[38]^	Female, 60–70 years	38/32	Stand upright with your hands on the handle	No intervention	30 Hz 0.5 mm	12 W	3	③
Santin-Medeiros et al. (2015)^[39]^	Female, 71–93 years	19/18	Stand, sit, squat, 18 exercises	No intervention	20 Hz 2 mm	32 W	2	①②⑤⑥
Shen et al. (2017)^[40]^	60–70 years old	Male 18, female 20/ Male 18, female 20	The knee joint is bent 110°−120°	No intervention	40 Hz 2 mm	8 W	3	②④
Song&Yang (2021)^[41]^	Female, 60–70 years	Low 19 Medium 18 High amplitude 19/20	Half squats, full squats, calf raises, and alternating single-leg squats	keep strokes	45 Hz 2 mm/3 mm /4 mm	24 W	3	②④⑤⑥
Von Stengel et al. (2009)^[42]^	Women aged 65 years or older	44/47	Heel raises, single-leg squats, leg abductions	Light exercise and relaxation	25–35 Hz 1.7 mm	48 W	2	①③
Von Stengel et al. (2011a)^[43]^	Female, 60–75 years	vvt34 rvt29/33	7 single-leg or double-leg dynamic leg exercises	Light exercise and relaxation	35 Hz 1.7 mm/ 12.5 Hz12 mm	48 W	3	②③
Von Stengel et al. (2011b)^[44]^	Women aged 65 years or older	46/48	Heel raises, single-leg squats, leg abductions	Light exercise and relaxation	25–35 Hz 1.7 mm	72 W	2	①③

① Total hip bone ② Femoral neck ③ Lumbar spine L1-L4 ④ Lumbar spine L2‒L4 ⑤ Ward’s triangle ⑥ Greater trochanter

### Methodological quality and risk of bias assessment

Table 2 presents the methodological quality assessment results for all TESTEX studies included in the review (The complete table is available in Supplement 4). Among these, 3 studies [36, 43, 44] demonstrated excellent quality, 5 studies [34, 35, 37, 38, 42] were rated as good quality, and 6 studies [31–33, 39–41] were rated as moderate quality. The average methodological quality score assessed by the TESTEX checklist was 9.5, indicating an overall good quality level.

**Table 2:** Methodological quality assessment questionnaire

study	Research quality					memoir											total points	class level
	1	2	3	4	5		6a	6b	6c	7	8a	8b	9	10	11	12		
Ba&Cheng (2016)^[31]^	1	0	0	1	0		0	0	0	1	1	0	1	1	1	1	8	Moderate
Ba&Cheng (2017)^[32]^	1	0	0	1	0		0	0	0	1	1	0	1	1	1	1	8	Moderate
Cheng et al. (2021)^[33]^	1	0	0	1	0		0	0	0	0	1	0	1	1	1	1	7	Moderate
Gómez-Cabello et al. (2014)^[34]^	1	0	0	1	0		1	1	0	1	1	1	1	0	1	1	10	Good
Gusi et al. (2006)^[35]^	1	0	0	0	0		1	1	1	0	1	1	1	1	1	1	10	Good
Leung et al. (2014)^[36]^	1	1	1	1	1		0	1	1	1	1	1	1	0	1	1	13	Excellent
Lu (2016)^[37]^	1	0	0	1	0		0	0	0	0	1	1	1	1	1	1	8	Good
Lu et al. (2012)^[38]^	1	0	0	1	0		1	1	0	1	1	1	1	0	1	1	10	Good
Santin-Medeiros et al. (2015)^[39]^	1	1	0	0	0		0	0	0	0	1	1	1	0	1	1	7	Moderate
Shen et al. (2017)^[40]^	1	0	0	1	0		0	0	0	1	1	1	1	0	1	1	8	Moderate
Song&Yang (2021)^[41]^	1	0	0	1	0		0	0	0	0	1	0	1	0	1	1	6	Moderate
Von Stengel et al. (2009)^[42]^	1	0	0	1	0		0	0	1	1	1	1	1	0	1	1	9	Good
Von Stengel et al. (2011a)^[43]^	1	1	1	1	1		0	1	1	1	1	1	1	1	1	1	14	Excellent
Von Stengel et al. (2011b)^[44]^	1	1	1	1	1		1	1	1	1	1	1	1	1	1	1	15	Excellent

(1) Eligibility; (2) Designated randomization; (3) Blinding of allocation; (4) Baseline similarity groups; (5) Assessors’ blinding; 6a. Participant compliance > 85%; 6b. Adverse events; 6c. Exercise adherence; 7. Intention-to-treat analysis; 8a. Primary outcome; 8b. Secondary outcomes; 9. Point estimates of all measured indicators; 10. Activity monitoring in the control group; 11. Relative exercise intensity was maintained; 12. Exercise volume and capacity expenditure

**Fig. 2:**
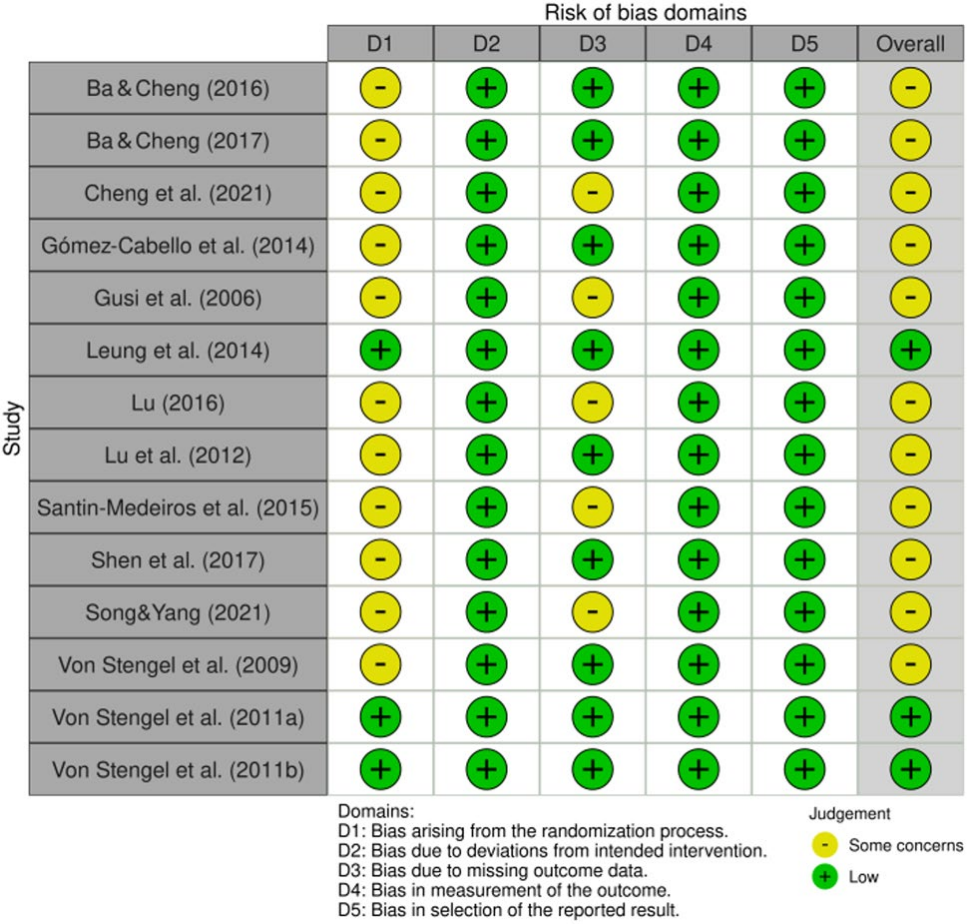
Summary of the risk of bias

**Fig. 3:**
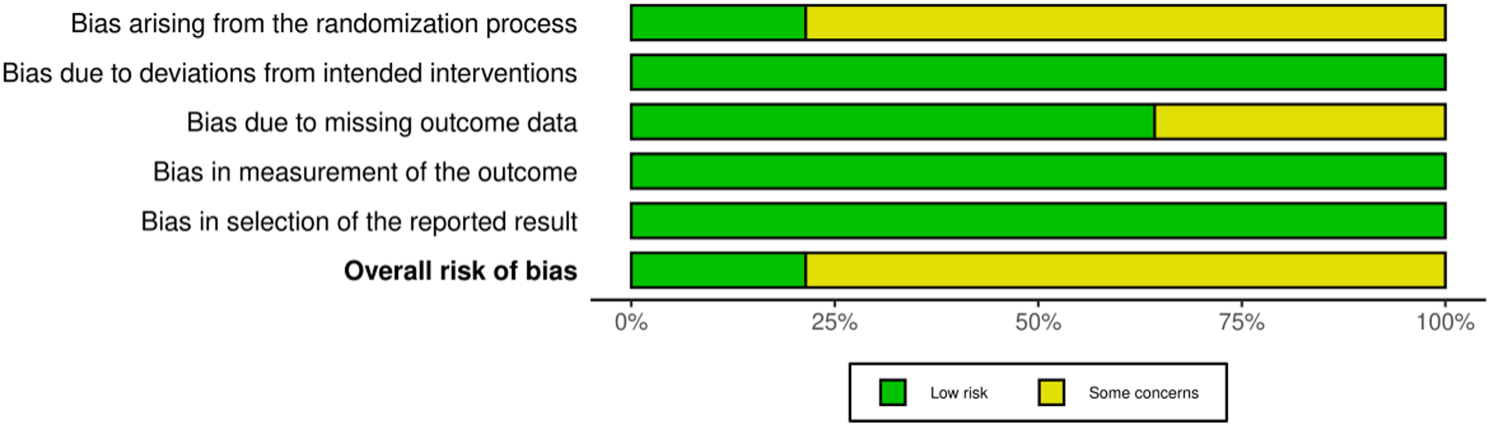
Risk of bias bar chart

Figures 2 and 3 present the Cochrane risk of bias assessments. Overall, 21% of studies [36, 43, 44] were rated as low risk, and 79% of studies [31–35, 37–42] were rated as having some concerns.

### Meta-analysis and sensitivity analysis

Fourteen included studies [31–44] conducted a comprehensive statistical analysis of bone mineral density values categorized across six sites—the femoral neck, Ward’s triangle region, greater trochanter, lumbar spine L1-L4, lumbar spine L2-L4, and total hip—to examine the effects of whole-body vibration training on bone mineral density at different sites in elderly populations.

### Effect of WBV on the outcome of the femoral neck

**Fig. 4:**
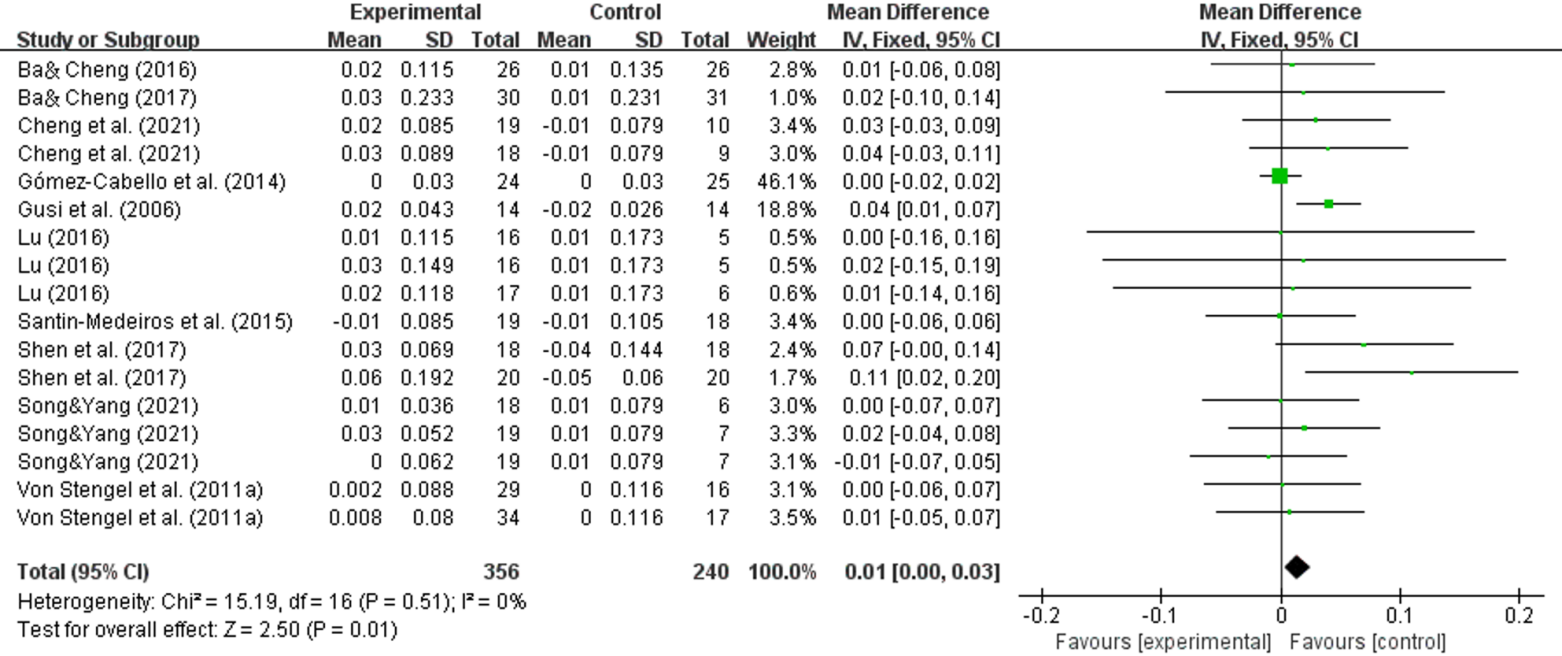
Effects of whole-body vibration training on the bone mineral density of the femoral neck in elderly individuals

A pooled analysis of 10 studies [31–35, 37, 39–41, 43] showed that whole-body vibration training improved femoral neck bone density in the elderly (WMD = 0.01, 95% CI (0.00–0.03), *P* = 0.01) (Fig. 4).

### Effect of WBV on the outcome of the Ward's triangle

**Fig. 5:**
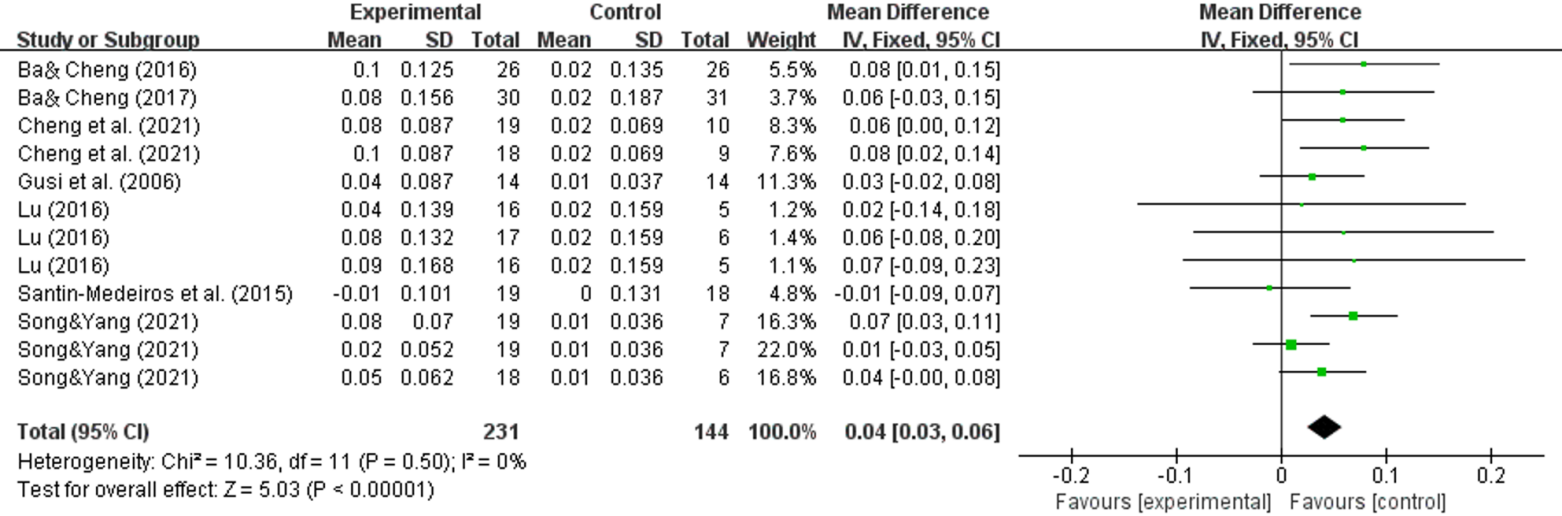
Effects of whole-body vibration training on the bone mineral density of the Ward's triangle in elderly individuals

In the Ward's triangle region, pooled results from 7 studies [31–33, 35, 37, 39, 41] demonstrated significant improvement (WMD = 0.04, 95% CI (0.03–0.06), P < 0.00001) (Fig. 5).

### Effect of WBV on the outcome of the greater trochanter

**Fig. 6:**
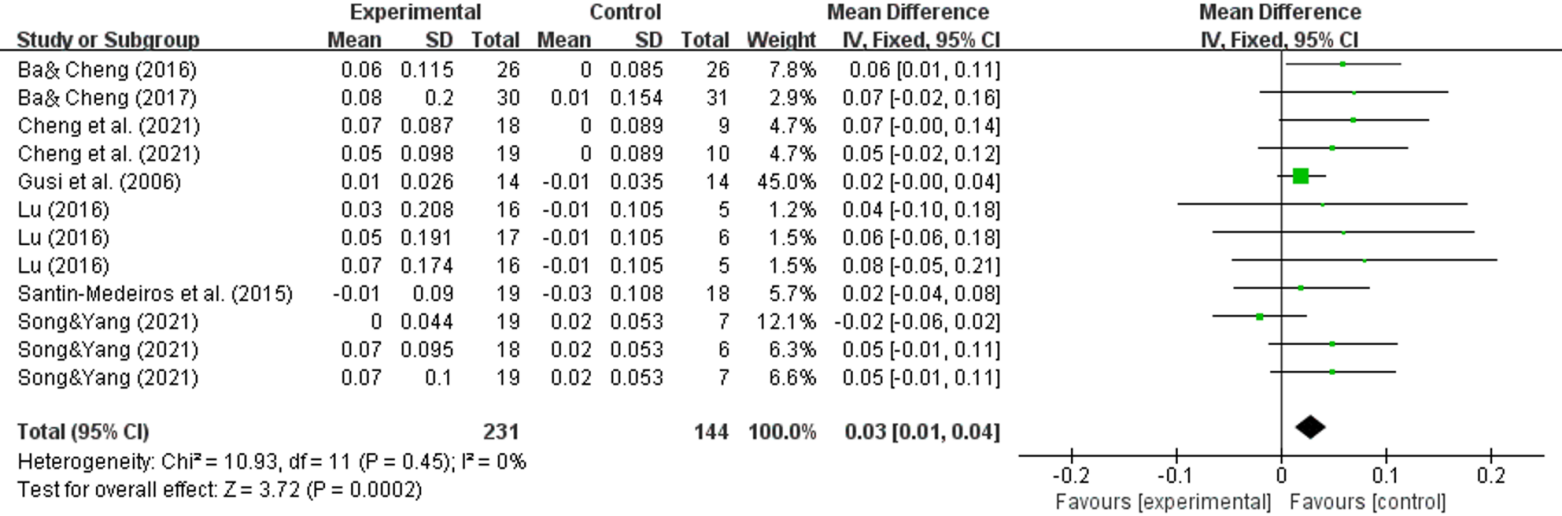
Effects of whole-body vibration training on the bone mineral density of the greater trochanter in elderly individuals

For the greater trochanter region, pooled results from 7 studies [31–33, 35, 37, 39, 41] also demonstrated significant bone density enhancement (WMD = 0.03, 95% CI (0.01–0.04), *P* = 0.0002) (Fig. 6).

### Effect of WBV on the outcome of the lumbar spine L2‒L4

**Fig. 7:**
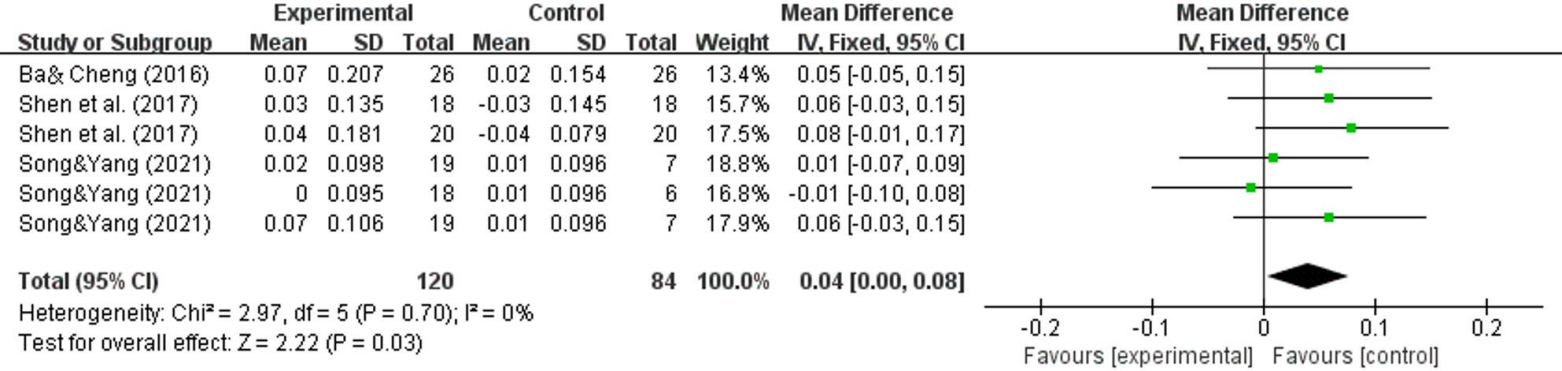
Effects of whole-body vibration training on the bone mineral density of the lumbar spine L2‒L4 in elderly individuals

A meta-analysis of three studies[31, 40, 41] revealed that whole-body vibration training significantly improved bone mineral density in the L2-L4 lumbar spine and femoral neck regions of elderly subjects (WMD = 0.04, 95% CI (0.00–0.08.00.08), *P* = 0.03) (Fig. 7).

### Effect of WBV on the outcome of the lumbar spine L1‒L4

**Fig. 8:**
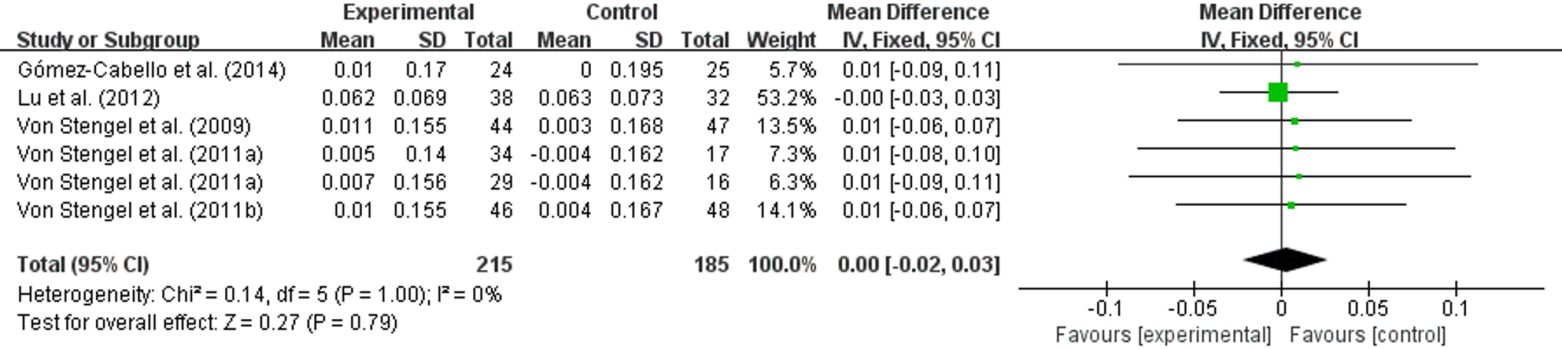
Effects of whole-body vibration training on the bone mineral density of the lumbar spine L1‒L4 in elderly individuals

However, a meta-analysis of five studies[34, 38, 42–44] showed no statistically significant effect of whole-body vibration training on bone mineral density in the L1-L4 lumbar spine region of older adults (WMD = 0.00, 95% CI (−0.02 to 0.03), *P* = 0.79) (Fig. 8).

### Effect of WBV on the outcome of the total hip bone

**Fig. 9:**
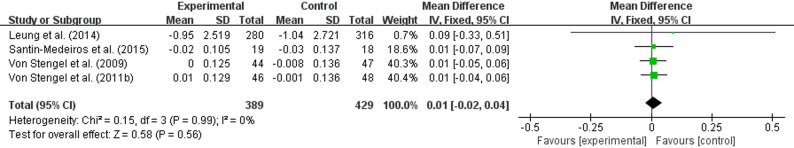
Effects of whole-body vibration training on the bone mineral density of the total hip bone in elderly individuals

Similarly, analysis of four studies[36, 39, 42, 44] found no statistically significant effect of whole-body vibration training on bone mineral density in the total hip region of older adults (WMD = 0.01, 95% CI (−0.02 to 0.04), *P* = 0.56) (Fig. 9).

To assess the impact of model selection on conclusions, we conducted a sensitivity analysis comparing results from fixed-effects and random-effects models. Findings indicate that effect estimates from both models share consistent directions, with substantial overlap in confidence intervals (Supplement 6), suggesting robust primary conclusions.

### Publication bias

Egger’s test was used to assess publication bias across studies. The results are presented in Table 3. No significant publication bias was detected for the femoral neck (*P* = 0.260), Ward’s triangle (*P* = 0.503), greater trochanter (*P* = 0.051), lumbar spine L2‒L4 (*P* = 0.738), lumbar spine L1‒L4 (*P* = 0.001), and total hip (*P* = 0.063). Publication bias was suspected only for the L1-L4 lumbar spine. The trim-and-trim method was applied, as shown in Table 4. After three studies were supplemented, the estimated effect size significantly increased, and the 95% confidence interval did not include zero, indicating statistical significance after trimming. However, the P value remained non-significant both before and after trimming. This suggests potential publication bias in the original meta-analysis.

**Table 3:** Egger test results for publication bias assessment

measuring point	Std_Eff	Coefficient	Std. err.	t	*P*>|t|	[95% conf. interval]
Femoral neck	Slope Bias	0.057	0.094	0.61	0.553	−0.014, 0.026
	Bias	0.459	0.392	1.17	0.260	−0.377, 1.294
Ward’s triangle	Slope Bias	0.297	0.021	1.45	0.179	−0.016, 0.076
	Bias	0.485	0.699	0.69	0.503	−1.072, 2.042
Greater trochanter	Slope Bias	0.003	0.013	0.24	0.813	−0.027, 0.033
	Bias	1.099	0.497	2.21	0.051	−0.008, 2.207
Lumbar spine L2-L4	Slope Bias	−0.063	0.289	−0.22	0.839	−0.866, 0.740
	Bias	2.291	6.385	0.36	0.738	−15.437, 20.019
Lumbar spine L1-L4	Slope Bias	−0.068	0.001	−4.87	0.008	−0.011, −0.003
	Bias	0.365	0.046	7.94	0.001	0.237, 0.493
Total hip bone	Slope Bias	−0.002	0.004	−0.58	0.622	−0.018, 0.014
	Bias	0.394	0.104	3.78	0.063	−0.055, 0.843

**Table 4:** Results of lumbar spine L1‒L4 splicing before and after surgery

Before and after cutting	Method	Est	95% CI	z price	*p* price	studies
before	Fixed/ Random	0.003	−0.021, 0.028	0.266	0.79	6
after		1.002	0.980, 1.024	0.151	0.88	9

### GRADE evidence quality assessment

The results of the GRADE assessment are presented in Table 5. The evidence quality grades are as follows: high-quality evidence for the femoral neck, Ward’s triangle, and greater trochanter regions; moderate-quality evidence for the lumbar spine L2‒L4, and total hip; and low-quality evidence for the lumbar spine L1‒L4.

**Table 5:** GRADE evidence quality assessment table

Quality assessment								No. patients patients patients		Effect	Quality	Importance
Outcomes	No of studies	Design	Risk of bias	Inconsistency	Indirectness	Imprecision	Other considerations	Trial	Control	Absolute Relative(95% CI)		
Femoral neck	10	randomized trials	no serious risk of bias	no serious inconsistency	no serious indirectness	no serious imprecision	none	356	240	0.01 [0 to 0.03]	HIGH	CRITICAL
Ward’s triangle	7	randomized trials	no serious risk of bias	no serious inconsistency	no serious indirectness	no serious imprecision	none	231	144	0.04[0.03 to 0.06]	HIGH	CRITICAL
Greater trochanter	7	randomized trials	no serious risk of bias	no serious inconsistency	no serious indirectness	no serious imprecision	none	231	144	0.03[0.01 to 0.04]	HIGH	CRITICAL
Lumbar spine L2-L4	3	randomized trials	no serious risk of bias	no serious inconsistency	no serious indirectness	serious1	none	120	84	0.04 [0 to 0.08]	MODERATE	CRITICAL
Lumbar spine L1-L4	5	randomized trials	no serious risk of bias	no serious inconsistency	no serious indirectness	serious3	reporting bias3	215	185	0 [−0.02 to 0.03]	LOW	CRITICAL
Total hip bone	4	randomized trials	no serious risk of bias	no serious inconsistency	no serious indirectness	serious3	none	389	429	0.01[−0.02 to 0.04]	MODERATE	CRITICAL

### Subgroup analysis

The subgroup analysis groups and results are shown in Table 6. Detailed forest plots are provided in Supplement 5. The vibration parameters not studied were ≤ 20 Hz and ≤ 2 mm, so this subgroup was not listed.

**Table 6:** Subgroup analysis

position		Vibration parameters					training cycle			action pattern	
		≤ 20 Hz >2 mm	20–40 Hz ≤ 2 mm	20–40 Hz>2 mm	≥ 40 Hz ≤ 2 mm	≥ 40 Hz >2 mm	< 24 weeks	24–36 weeks	> 36 weeks	static modes	dynamic modes
Femoral neck	①	4	2	3	4	4	3	12	2	4	13
	②	0%	0%	0%	67%	0%	77%	0%	0%	77%	0%
	③	0.03	0	0.01	0.01	0.02	0.01	0.02	0.01	0.02	0.01
	④	[0.01, 0.06]	[−0.04, 0.05]	[−0.04, 0.07]	[−0.01, 0.02]	[−0.02, 0.06]	[−0.01, 0.02]	[0.01, 0.04]	[−0.04, 0.05]	[0, 0.03]	[−0.01, 0.03]
	⑤	0.004	0.86	0.66	0.45	0.29	0.39	0.005	0.82	0.02	0.28
Ward’s triangle	①	3	1	3	1	4	0	12	0	1	11
	②	0%		0%		0%		0%			1%
	③	0.04		0.07		0.06		0.04			0.04
	④	[0, 0.08]		[0.02, 0.12]		[0.03, 0.09]		[0.03, 0.06]			[0.03, 0.06]
	⑤	0.03		0.007		<0.001		<0.001			<0.001
Greater trochanter	①	3	1	3	1	4	0	12	0	1	11
	②	0%		0%		0%		0%			0%
	③	0.02		0.06		0.06		0.03			0.04
	④	[0, 0.04]		[0.02, 0.11]		[0.02, 0.09]		[0.01, 0.04]			[0.02, 0.06]
	⑤	0.03		0.005		0.001		0.0002			0.005
Lumbar spine L2-L4	①	0	0	1	3	2	2	4	0	2	4
	②				0%	19%	0%	0%		0%	0%
	③				0.05	0.03	0.07	0.03		0.07	0.03
	④				[−0, 0.1]	[−0.04, 0.09]	[0.01, 0.13]	[−0.02, 0.07]		[0.01, 0.13]	[−0.02, 0.07]
	⑤				0.06	0.46	0.03	0.24		0.03	0.24
Lumbar spine L1-L4	①	1	4	0	1	0	2	0	4	2	4
	②		0%				0%		0%	0%	0%
	③		0				0		0.01	0	0.01
	④		[−0.02, 0.03]				[−0.03, 0.03]		[−0.03, 0.05]	[−0.03, 0.03]	[−0.03, 0.05]
	⑤		0.86				1		0.68	1	0.68
Total hip bone	①	0	4	0	0	0	0	2	2	1	3
	②		0%					0%	0%		0%
	③		0.01					0.01	0.01		0.01
	④		[−0.02, 0.04]					[−0.06, 0.09]	[−0.03, 0.05]		[−0.02, 0.04]
	⑤		0.56					0.75	0.62		0.58

The blank space is due to an insufficient sample size, which does not meet the minimum requirements for subgroup analysis. ① Number of studies; ② I^2^; ③ Effect size; ④ 95% CI; ⑤ P value

### Vibration parameters

Subgroup analysis based on vibration parameters (frequency, amplitude) revealed the following: frequency ≤ 20 Hz and amplitude > 2 mm: the femoral neck, Ward’s triangle, and greater trochanter met the subgroup criteria, resulting in significant improvement (I^2^ = 0%, *P* ≤ 0.03, 95% CI [0, 0.08]).

For frequencies ranging from 20 to 40 Hz and amplitudes ≤ 2 mm, the femoral neck, lumbar spine L1–L4, and total hip met the subgroup criteria but showed nonsignificant changes (I^2^ = 0%, *P* > 0.05, 95% CI [− 0.04, 0.05]). For frequencies ranging from 20 to 40 Hz with amplitudes > 2 mm, the femoral neck, Ward’s triangle region, and greater trochanter met the subgroup criteria, with significant improvement observed in the Ward’s triangle region and greater trochanter (I^2^ = 0%, *P* < 0.07, 95% CI [0.02, 0.12]).

For frequency ≥ 40 Hz, amplitude ≤ 2 mm: Only the femoral neck and lumbar spine L2-L4 met subgroup criteria, but neither showed significant efficacy (I² ≥0%, *P* > 0.05, 95% CI [−0.01, 0.1]). For frequency ≥ 40 Hz and amplitude > 2 mm, the femoral neck, Ward’s triangle region, greater trochanter, and lumbar spine L2-L4 met subgroup requirements. Only the Ward’s triangle region and greater trochanter showed significant effects (I²=0%, *P* < 0.001, 95% CI [0.02, 0.09]).

### Training cycle

Subgroup analysis results for training duration: During 24–36 weeks of intervention, significant improvements were observed in the femoral neck, Ward’s triangle region, and greater trochanter (I^2^ = 0%, *P* < 0.005, 95% CI [0.01, 0.04]), except for the lumbar spine L1‒L4, which failed to meet subgroup criteria.

Interventions at < 24 weeks, the femoral neck, lumbar spine L1‒L4, and lumbar spine L2‒L4 met subgroup requirements, with improvements observed only in lumbar spine L2‒L4 (I^2^ = 0%, *P* = 0.03, 95% CI [0.01, 0.13]).

Interventions at > 36 weeks met analysis requirements only for the femoral neck, L1–L4 lumbar spine, and total hip, but no significant changes were observed (I^2^ = 0%, *P* ≥ 0.62, 95% CI [−0.04, 0.05]).

### Movement patterns

The movements are classified into static modes (standing, static squat) and dynamic modes (half-squat, heel raise, single-leg squat, deep squat, etc.). Subgroup analysis revealed that dynamic modes significantly improved BMD in Ward’s triangle and the greater trochanter (I²≤ 1%, *P* ≤ 0.005, 95% CI [0.02, 0.06]).

For the static mode, only the femoral neck, lumbar spine L1‒L4, and lumbar spine L2‒L4 met the subgroup requirements. Specifically, the femoral neck (I² = 77%, *P* < 0.00001, 95% CI [0.00, 0.08]) and lumbar spine L2‒L4 (I^2^ = 0%, *P* = 0.03, 95% CI [0.01, 0.13]) improved significantly.

## Discussion

### Meta-analysis results and quality assessment

Combining evidence quality grades with meta-analysis results further indicates that whole-body vibration training substantially increases bone mineral density in the Ward’s triangle and greater trochanter regions of elderly individuals, slightly increases it in the femoral neck, may substantially increase it in the lumbar spine L2‒L4, and has negligible effects on the lumbar spine L1‒L4 and total hip.

No significant heterogeneity was detected across sites. It is possible that by selecting the “difference between pre-treatment and post-treatment measurements” as the composite outcome measure in this study, the strategy has to some extent controlled for baseline heterogeneity. Furthermore, the sample sizes or statistical power of the currently included studies may be insufficient to detect subtle differences in effect sizes arising from clinical variations. However, potential publication bias exists for the lumbar spine L1‒L4, with possible sources including: bias from deviation from the intended intervention, and reduced statistical power due to an insufficient sample size.

The limitations in reporting exercise protocols and sufficient detail in the TESTEX scores compensate for the shortcomings in bias risk assessment. A comparative analysis of TESTEX scores versus bias risk assessments [31–33, 39–41] revealed that all six studies scored relatively low. Key deficiencies included inadequate documentation of randomization procedures and blinding methods, as well as insufficient reporting of intervention details. These shortcomings may have influenced study outcomes, necessitating cautious interpretation of results.

### Site-specific responsiveness

From a human anatomical perspective, the variations across different body regions primarily stem from the nonlinear characteristics of the musculoskeletal system and the differing receptivity of body segments to stimulus intensity during WBVT. Significant disparities exist in mechanical transduction efficiency among body areas, resulting in uneven distributions and intensities of mechanical stimulation across regions [45].

For example, during vertical sinusoidal vibration, peak acceleration may substantially increase at frequencies of 10–40 Hz for the ankle joint, 10–25 Hz for the knee joint, and 10 Hz for the spine. Beyond these thresholds, the transmitted vibrational power decreases to approximately one-tenth of the platform’s power output. The hip joint is at 10–20 Hz, and the spine is at 10 Hz. Beyond these frequencies, the transmitted vibration power decreases to 1/10 to 1/1000 of the platform’s delivered power [45].

The results of this study indicate significant effects on Ward’s triangle and the greater trochanter region. On the one hand, as noted by Gusi et al. [35], when the lateral acceleration of the vibration platform exceeds the vertical acceleration, causing one half of the platform to rise while the other half descends, the hips must maintain continuous balance, resulting in greater stimulation to the hip region.

On the other hand, WBVT transmits mechanical energy from the platform to the body, increasing skeletal mechanical loading and stimulating bone cell metabolism to increase bone density. However, WBVT generates a nonuniform stress distribution. Studies have indicated that the tensile stress on the lateral upper femoral neck consistently exceeds the compressive stress on the medial lower neck. As the trabecular bone volume fraction decreases, both tensile and compressive stresses on the proximal femur progressively increase [46]. This may explain the significant improvement in BMD observed in Ward’s triangle and the greater trochanter.

The substantial increase in lumbar effects may be related to the load characteristics of the lumbar region. Von Stengel et al. [43] noted that the upward load frequency in the pelvic region is twice that of each leg. Compared with the entire hip, this higher load frequency may subject the spinal bones to stronger mechanical stimulation, thereby increasing the bone density in the lumbar region. Owing to their relatively lower position and greater mechanical loading, the mechanical conductivity of lumbar vertebrae L2‒L4 is greater than that of L1‒L4.

Only Gusi et al. [35] and Shen Yanmei et al. [40] reported significant effects of WBVT on the femoral neck. Potential reasons for the limited efficacy in the femoral neck region include: Lu Pengtao et al. [37], Cheng et al. [33], and Song et al. [41], who suggested that the intervention duration was insufficient to achieve adequate cumulative effects for improving bone density. Additionally, Ba Hongbing et al. [31, 32] suggested that lower vibration frequencies or insufficient stimulation intensity may contribute to these findings.

The specific mechanisms underlying the limited efficacy in the femoral neck region remain incompletely elucidated and warrant further investigation. Differences in skeletal responses to WBVT suggest that the response characteristics of target bone regions should be considered when designing intervention protocols.

### Vibration parameters

The vibration parameters include the vibration frequency and vibration amplitude. Currently, there is no unified standard for optimal parameters in whole-body vibration therapy (WBVT) for elderly individuals. Most research parameters align with Abazović et al.‘s recommendations, utilizing frequencies between 20 and 40 Hz and amplitudes ranging from 2.0 to 5.0 mm, administered for up to 30 min daily, three times weekly [47].

With respect to vibration frequency, subgroup analysis revealed that all frequency bands significantly improved BMD in the Ward’s triangle and greater trochanter regions. Lu Pengtao et al. [37] compared WBVT at different frequencies but identical amplitudes and reported that both medium- and high-frequency vibrations significantly increased BMD in the elderly individuals, with high-frequency training yielding superior outcomes compared with low- and medium-frequency regimens. This finding aligns with the findings of Kim et al., who demonstrated that daily vibration stimulation promotes the proliferation of bone marrow stromal cells, achieving optimal efficiency within the 30–40 Hz frequency range [48].

Subgroup analysis also revealed that vibration frequencies ≤ 20 Hz significantly increased femoral neck BMD. Gusi et al. [35] reported that low-amplitude (3 mm) moderate-frequency (12.6 Hz) vibration loading can prevent age-related hip bone loss, particularly in frail individuals. Additionally, Camacho-Cardenosa et al. [49] demonstrated significant effects on the proximal femur of elderly individuals using the same 12.6 Hz frequency combined with hypoxic training, highlighting the safe and noninvasive nature of this protocol.

With respect to vibration amplitude, subgroup analysis revealed that amplitudes > 2 mm were more conducive to enhancing BMD. Song et al. [41] compared the effects of low-amplitude (2 mm), medium-amplitude (3 mm), and high-amplitude (4 mm) WBVT on BMD in elderly individuals. The results revealed a positive correlation between amplitude and BMD gains, suggesting the consideration of high-amplitude WBVT. At constant frequency, the eccentric contraction force in the vibrating muscles increases with amplitude, potentially increasing bone surface stress levels. Massini et al. [25] also proposed that increasing the vibration amplitude could generate higher peak acceleration values at frequencies below 50 Hz.

Notably, while existing studies have not reported vibration-related adverse reactions, high-intensity WBVT may cause negative effects such as dizziness, headaches, and falls, potentially damaging peripheral nerves and blood vessels [50].

Based on current evidence, WBVT parameters can be tailored to individual health conditions in older adults: higher frequencies (e.g., 30–40 Hz) may be considered for those in good health, while lower frequencies (e.g., 10–15 Hz) combined with other rehabilitation measures may be more suitable for frail individuals. Regarding vibration amplitude, exploratory studies suggest that amplitudes exceeding 2 mm may yield more positive effects. Such personalized parameter adjustment strategies hold promise for enhancing the overall efficacy of WBVT while mitigating potential risks.

### Training cycles

The training cycle is one of the key factors influencing the efficacy of WBVT. Subgroup analysis revealed that when the training cycle was < 24 weeks, WBVT produced significant improvements in BMD only in the lumbar spine region (L2-L4).

This finding aligns with the research conclusions of Gómez-Cabello et al. [34], indicating that short-term WBVT induces only minor changes in elderly skeletal structure, with BMD requiring at least 6 months or longer to demonstrate a significant response. When the training period was extended to 24–36 weeks, the increase in BMD values was most pronounced. Concurrently, research by Bahongbing et al. [32] demonstrated that after discontinuing 24 weeks of WBVT were discontinued, improvements in greater trochanter BMD persisted for 8 weeks post-cessation. However, the BMD in Ward’s triangle region decreased, suggesting the potential loss of increased BMD and indicating that continuous WBVT plays a positive role in maintaining BMD.

However, subgroup analysis revealed no significant improvement in BMD when the intervention period exceeded 36 weeks. Leung et al. [36] reported that bone effects may require longer stimulation periods (at least 9 months) and more frequent interventions (i.e., vibration more than 4 times per week). While BMD differences in older adults after WBVT may not be significant initially, positive trends, such as reduced fall fracture rates, emerge later. Theoretically, bone formation, resorption, and mineralization processes require 3–4 months, whereas bone mass reaches a new steady state in approximately 6–8 months [51]. In summary, a WBVT regimen lasting ≥ 24 weeks with a high intervention frequency may be reasonable.

### Movement patterns

Previous studies have demonstrated that mechanical vibration can induce physiological changes at multiple levels, improving neuromuscular function through postural control strategies, muscle adjustment mechanisms, and tonic vibration reflexes [52]. Consequently, the movement pattern of whole-body vibration therapy (WBVT) is also a key factor influencing BMD. Subgroup analysis revealed that the dynamic pattern significantly improved the BMD in the Ward’s triangle and greater trochanter regions, whereas the static pattern increased the BMD in the femoral neck and lumbar spine L2‒L4.

The mechanism underlying the dynamic pattern’s enhancement of hip BMD is twofold. First, when subjects maintain an upright posture, 30 Hz mechanical vibration achieves 70% transmission efficiency to the lower limbs and spine [53]. However, increasing the hip–knee–ankle flexion angle correspondingly reduces vibration transmission at the spine while elevating lower limb muscle activation levels. Hip joint vibration acceleration peaks during flexion (150–165°) [54]. Vibration stimulation and muscle contraction synergistically enhance the hip effect.

Additionally, during dynamic squatting movements, the descent and ascent phases correspond to lower limb flexion and extension, respectively. The rectus femoris, vastus medialis, and vastus lateralis primarily contribute to hip flexion and knee extension, whereas the gluteus maximus, biceps femoris, and gastrocnemius mainly govern hip extension and knee flexion [55]. During muscle activity, external vibration stimulation significantly enhances activation levels in the thigh muscle groups. Such neuromuscular adaptations may positively influence BMD.

Static modes are primarily categorized as standing and static squatting. Von Stengel et al. [43] suggested that when participants stand vertically, placing body weight on the heels during vibration exposure can increase stress transmission along the spinal axis, thereby increasing lumbar BMD.

However, standing vibration may induce discomfort such as dizziness, potentially due to lower damping effects resulting in higher vibration doses to the upper body [54]. Appropriate knee flexion can mitigate this discomfort. Shen Yanmei et al. [40] confirmed that the mineral density of the hip bone is significantly greater than that of the lumbar spine in a static knee-bent posture, potentially related to vibration energy attenuation in the spinal region due to lower limb flexion.

Further clinical research is needed to elucidate the mechanisms and intervention effects of dynamic versus static movement patterns, as well as upright versus lower-limb-flexed postures, on BMD in older adults during WBVT. Based on existing exploratory evidence, the following targeted training approaches may be considered: For enhancing lumbar bone density, static training modes may hold greater potential; for improving hip bone density, dynamic training modes may be the superior choice. Notably, static training modes also demonstrate some positive effects on hip bone density.

### Limitations

These studies have certain limitations, and the reliability of the findings may be influenced by multiple factors.

1. Few studies included males, with most focusing on females, potentially introducing gender bias.
2. Despite conducting a comprehensive literature search, access to certain unpublished data was limited—to maintain methodological transparency and reproducibility, the study primarily relied on publicly available literature for analysis, which may have impacted the completeness of the results to some extent.
3. The small number of included studies resulted in insufficient power to detect effects at the lumbar L2–L4, L1–L4, and total hip sites.
4. Incomplete reporting in some included studies increased the difficulty in quality assessment and risk of bias.
5. Potential publication bias in some studies.
6. small sample sizes in certain studies limited the detection of true intervention effects.
7. insufficient studies for comprehensive subgroup analysis. Collectively, these factors restrict the generalizability of the findings to the elderly population.

## Conclusions

Whole-body vibration training significantly increased bone mineral density in Ward’s triangle and greater trochanter regions in elderly subjects, with smaller improvements in the femoral neck region. The L2‒L4 lumbar spine segment may substantially increase, whereas the L1‒L4 lumbar spine segment and total hip region have negligible effects. Future research should further explore the effects of whole-body vibration training on bone mineral density in elderly men, and delve into the response characteristics of different body regions to this training method and its underlying mechanisms.

## Supplementary Information

Supplementary Material 1.

Supplementary Material 2.

Supplementary Material 3.

Supplementary Material 4.

Supplementary Material 5.

Supplementary Material 6.

## Data Availability

All data generated or analysed during this study are included in this published article.
