# Supplementary material for: Whole-body vibration training and bone mineral density in older adults: an updated systematic review and meta-analysis
Source: BMC Musculoskelet Disord. 2026 Jan 21;27:149. doi: 10.1186/s12891-026-09504-7 (PMC12908257; doi:10.1186/s12891-026-09504-7)
Supplement: Supplementary file 1 — Supplementary Material 1. [file 12891_2026_9504_MOESM1_ESM.docx]

**Supplement 1. Search strategy to whole-body vibration training and bone mineral density in older adults**

CNKI

| Step | Search terms | # of publications |
| --- | --- | --- |
| 1 | (SU%=‘elderly’ OR SU%=‘senior’) AND (SU%=‘vibration’ OR SU%=‘whole-body vibration’ OR SU%=‘vibration training’) AND (SU%=‘bone mineral density’ OR SU%=‘bone density’ OR SU%=‘osteoporosis’) | 61 |

VIP Chinese Journal Database

| Step | Search terms | # of publications |
| --- | --- | --- |
| 1 | M=(Aging OR Elderly) AND M=(Vibration OR Whole-Body Vibration OR Vibration Training) AND M=(Bone Mineral Density OR Osteoporosis OR Bone Density) | 22 |

Wanfang Database

| Step | Search terms | # of publications |
| --- | --- | --- |
| 1 | (Subject:(Elderly) or Subject:(Aging)) and (Subject:(Vibration) or Subject:(Whole-body vibration) or Subject:(Vibration training)) and (Subject:(Bone density) or Subject:(Osteoporosis) or Subject:(Bone mineral density)) | 71 |

Chinese Biomedical Literature Database

| Step | Search terms | # of publications |
| --- | --- | --- |
| 1  2  3  4  5  6  7  8  9  10  11  12 | “Elderly”[unweighted:expanded]  Elderly  (Elderly) OR (“Elderly”[unweighted:expanded])  “Vibration”[unweighted:extended]  Whole-body vibration  Vibration Training  (Vibration Training) OR (Whole-body Vibration) OR (“Vibration”[unweighted:extended])  “Bone density”[unweighted:expanded]  Bone Mineral Density  Osteoporosis  (Osteoporosis) OR (Bone Mineral Density) OR (“Bone Density”[unweighted:expanded])  ((Osteoporosis) OR (Bone Mineral Density) OR (“Bone Density”[unweighted:expanded])) AND ((Vibration Training) OR (Whole-Body Vibration) OR (‘Vibration’[unweighted:expanded])) AND ((Elderly) OR (“Elderly”[unweighted:expanded])) | 50854  937477  985650  9424  374  295  9484  32963  134018  191941  254869  41 |

PubMed

| Step | Search terms | # of publications |
| --- | --- | --- |
| 1 | ((("Aged"[Mesh]) OR (((((((Elderly[Title/Abstract]) OR (ageing[Title/Abstract])) OR (Senior[Title/Abstract])) OR (Aging[Title/Abstract])) OR (Old[Title/Abstract])) OR (Elder[Title/Abstract])) OR (older[Title/Abstract]))) AND (("Vibration"[Mesh]) OR (((((((((Vibrations[Title/Abstract]) OR (whole body vibration training[Title/Abstract])) OR (WBV[Title/Abstract])) OR (WBVT[Title/Abstract])) OR (whole body vibration[Title/Abstract])) OR (vibration exercise[Title/Abstract])) OR (vibration training[Title/Abstract])) OR (vibration therapy[Title/Abstract])) OR (Vibratory[Title/Abstract])))) AND (("Bone Density"[Mesh]) OR ((((((((((((((((((((((((Bone Densities[Title/Abstract]) OR (Density, Bone[Title/Abstract])) OR (Bone Mineral Densities[Title/Abstract])) OR (Bone Mineral Density[Title/Abstract])) OR (Bone Mineral Contents[Title/Abstract])) OR (Bone Mineral Content[Title/Abstract])) OR (Osteoporosis[Title/Abstract])) OR (Osteoporoses[Title/Abstract])) OR (Osteoporosis, Age-Related[Title/Abstract])) OR (Osteoporosis, Age Related[Title/Abstract])) OR (Age-Related Osteoporosis[Title/Abstract])) OR (Age-Related Osteoporoses[Title/Abstract])) OR (Age Related Osteoporosis[Title/Abstract])) OR (Osteoporoses, Age-Related[Title/Abstract])) OR (Bone Loss, Age-Related[Title/Abstract])) OR (Age-Related Bone Loss[Title/Abstract])) OR (Age-Related Bone Losses[Title/Abstract])) OR (Bone Loss, Age Related[Title/Abstract])) OR (Bone Losses, Age-Related[Title/Abstract])) OR (Osteoporosis, Senile[Title/Abstract])) OR (Osteoporoses, Senile[Title/Abstract])) OR (Senile Osteoporoses[Title/Abstract])) OR (Senile Osteoporosis[Title/Abstract])) OR (Osteoporosis, Involutional[Title/Abstract]))) | 212 |

Cochrane Library

| Step | Search terms | # of publications |
| --- | --- | --- |
| 1  2  3  4  5  6  7  8  9  10  11  12  13  14  15  16 | MeSH descriptor: [Aged] explode all trees  (Elderly):ti,ab,kw OR (ageing):ti,ab,kw OR (Senior):ti,ab,kw OR (Aging):ti,ab,kw OR (Old):ti,ab,kw (Word variations have been searched)  (Elder):ti,ab,kw OR (Older):ti,ab,kw (Word variations have been searched)  #1 or #2 or #3  MeSH descriptor: [Vibration] explode all trees  (Vibrations):ti,ab,kw OR (whole body vibration training):ti,ab,kw OR (WBV):ti,ab,kw OR (WBVT):ti,ab,kw OR (whole body vibration):ti,ab,kw (Word variations have been searched)  (vibration exercise):ti,ab,kw OR (vibration training):ti,ab,kw OR (vibration therapy):ti,ab,kw OR (Vibratory):ti,ab,kw (Word variations have been searched)  #5 or #6 or #7  MeSH descriptor: [Bone Density] explode all trees  (Bone Densities):ti,ab,kw OR (Density, Bone):ti,ab,kw OR (Bone Mineral Densities):ti,ab,kw OR (Bone Mineral Density):ti,ab,kw OR (Bone Mineral Contents):ti,ab,kw (Word variations have been searched)  (Bone Mineral Content):ti,ab,kw OR (Osteoporosis):ti,ab,kw OR (Osteoporoses):ti,ab,kw OR (Osteoporosis, Age-Related):ti,ab,kw OR (Osteoporosis, Age Related):ti,ab,kw (Word variations have been searched)  (Age-Related Osteoporosis):ti,ab,kw OR (Age-Related Osteoporoses):ti,ab,kw OR (Age Related Osteoporosis):ti,ab,kw OR (Osteoporoses, Age-Related):ti,ab,kw OR (Bone Loss, Age-Related):ti,ab,kw (Word variations have been searched)  (Age-Related Bone Loss):ti,ab,kw OR (Age-Related Bone Losses):ti,ab,kw OR (Bone Loss, Age Related):ti,ab,kw OR (Bone Losses, Age-Related):ti,ab,kw OR (Osteoporosis, Senile):ti,ab,kw (Word variations have been searched)  (Osteoporoses, Senile):ti,ab,kw OR (Senile Osteoporoses):ti,ab,kw OR (Senile Osteoporosis):ti,ab,kw OR (Osteoporosis, Involutional):ti,ab,kw (Word variations have been searched)  #9 or #10 or #11 or #12 or #13 or #14  #4 and #8 and #15 | 277139  1010343  132577  1022298  1602  5403  3880  5607  5970  15991  13599  2261  1843  167  22513  202 |

EMBASE

| Step | Search terms | # of publications |
| --- | --- | --- |
| 1  2  3  4  5  6  7  8  9  10  11  12  13  14  15  16  17  18  19  20  21  22  23  24  25  26  27  28  29  30  31  32  33  34  35  36  37  38  39  40  41  42  43  44  45  46  47 | 'aged'/exp OR 'aged'  'elderly':ti,ab  'ageing':ti,ab  'senior':ti,ab  'aging':ti,ab  'old':ti,ab  'elder':ti,ab  'older':ti,ab  #1 OR #2 OR #3 OR #4 OR #5 OR #6 OR #7 OR #8  'vibration'/exp OR 'vibration'  'vibrations':ti,ab  'whole body vibration training':ti,ab  'wbv':ti,ab  'wbvt':ti,ab  'whole body vibration':ti,ab  'vibration exercise':ti,ab  'vibration training':ti,ab  'vibration therapy':ti,ab  'vibratory':ti,ab  #10 OR #11 OR #12 OR #13 OR #14 OR #15 OR #16 OR #17 OR #18 OR #19  'bone density'/exp OR 'bone density'  'bone densities':ti,ab  'bone mineral densities':ti,ab  'density, bone':ti,ab  'bone mineral density':ti,ab  'bone mineral contents':ti,ab  'bone mineral content':ti,ab  'osteoporosis':ti,ab  'osteoporoses':ti,ab  'osteoporosis, age-related':ti,ab  'osteoporosis, age related':ti,ab  'age-related osteoporosis':ti,ab  'age-related osteoporoses':ti,ab  'age related osteoporosis':ti,ab  'osteoporoses, age-related':ti,ab  'bone loss, age-related':ti,ab  'age-related bone loss':ti,ab  'age-related bone losses':ti,ab  'bone loss, age related':ti,ab  'bone losses, age-related':ti,ab  'osteoporosis, senile':ti,ab  'osteoporoses, senile':ti,ab  'senile osteoporoses':ti,ab  'senile osteoporosis':ti,ab  'osteoporosis, involutional':ti,ab  #21 OR #22 OR #23 OR #24 OR #25 OR #26 OR #27 OR #28 OR #29 OR #30 OR #31 OR #32 OR #33 OR #34 OR #35 OR #36 OR #37 OR #38 OR #39 OR #40 OR #41 OR #42 OR #43 OR #44 OR #45  #9 AND #20 AND #46 | 6,489,860  442,713  77,233  67,745  326,945  2,045,885  16,458  873,350  8,169,945  130,065  16,843  444  2,266  123  3,114  345  643  387  5,327  138,586  134,283  809  1,075  1,575  74,825  187  9,703  135,066  182  12  12  367  2  367  0  3  1,108  2  3  0  12  0  3  930  0  225,853  399 |

Web of Science

| Step | Search terms | # of publications |
| --- | --- | --- |
| 1  2  3  4 | (((((((TS=(aged)) OR TS=(elderly)) OR TS=(ageing)) OR TS=(senior)) OR TS=(aging)) OR TS=(old)) OR TS=(elder)) OR TS=(older)  Timespan: All Years (Publication Date)  (((((((((TS=(vibration)) OR TS=(vibrations)) OR TS=(whole body vibration training)) OR TS=(WBV)) OR TS=(WBVT)) OR TS=(whole body vibration)) OR TS=(vibration exercise)) OR TS=(vibration training)) OR TS=(vibration therapy)) OR TS=(vibratory)  ((((((((((((((((((((((((TS=(Bone Density)) OR TS=(Bone Densities)) OR TS=(Density, Bone)) OR TS=(Bone Mineral Densities)) OR TS=(Bone Mineral Density)) OR TS=(Bone Mineral Contents)) OR TS=(Bone Mineral Content)) OR TS=(Osteoporosis)) OR TS=(Osteoporoses)) OR TS=(Osteoporosis, Age-Related)) OR TS=(Osteoporosis, Age Related)) OR TS=(Age-Related Osteoporosis)) OR TS=(Age-Related Osteoporoses)) OR TS=(Age Related Osteoporosis)) OR TS=(Osteoporoses, Age-Related)) OR TS=(Bone Loss, Age-Related)) OR TS=(Age-Related Bone Loss)) OR TS=(Age-Related Bone Losses)) OR TS=(Bone Loss, Age Related)) OR TS=(Bone Losses, Age-Related)) OR TS=(Osteoporosis, Senile)) OR TS=(Osteoporoses, Senile)) OR TS=(Senile Osteoporoses)) OR TS=(Senile Osteoporosis)) OR TS=(Osteoporosis, Involutional)  #3 AND #2 AND #1 | 15013894  1846427  446457  979 |
