# Supplementary material for: Whole-body vibration training and bone mineral density in older adults: an updated systematic review and meta-analysis
Source: BMC Musculoskelet Disord. 2026 Jan 21;27:149. doi: 10.1186/s12891-026-09504-7 (PMC12908257; doi:10.1186/s12891-026-09504-7)
Supplement: Supplementary file 2 — Supplementary Material 2. [file 12891_2026_9504_MOESM2_ESM.docx]

**Supplement 2 Data summary for each intervention group**

| Measurement site | Study | Year | Tsample | Tmean | Tsd | Csample | Cmean | Csd |
| --- | --- | --- | --- | --- | --- | --- | --- | --- |
| Femoral neck | Ba＆Cheng | 2016 | 26 | 0.02 | 0.115 | 26 | 0.01 | 0.135 |
|  | Ba＆Cheng | 2017 | 30 | 0.03 | 0.233 | 31 | 0.01 | 0.231 |
|  | Shen et al. | 2017 | 18 | 0.03 | 0.069 | 18 | -0.04 | 0.144 |
|  | Shen et al. | 2017 | 20 | 0.06 | 0.192 | 20 | -0.05 | 0.06 |
|  | Lu | 2016 | 16 | 0.01 | 0.115 | 5 | 0.01 | 0.173 |
|  | Lu | 2016 | 17 | 0.02 | 0.118 | 6 | 0.01 | 0.173 |
|  | Lu | 2016 | 16 | 0.03 | 0.149 | 5 | 0.01 | 0.173 |
|  | Gómez-Cabello,A | 2014 | 24 | 0 | 0.03 | 25 | 0 | 0.03 |
|  | Gusi, N | 2006 | 14 | 0.02 | 0.043 | 14 | -0.02 | 0.026 |
|  | Santin-Medeiros, F | 2015 | 19 | -0.01 | 0.085 | 18 | -0.01 | 0.105 |
|  | Song&Yang | 2021 | 19 | 0 | 0.062 | 7 | 0.01 | 0.079 |
|  | Song&Yang | 2021 | 18 | 0.01 | 0.036 | 6 | 0.01 | 0.079 |
|  | Song&Yang | 2021 | 19 | 0.03 | 0.052 | 7 | 0.01 | 0.079 |
|  | Von Stengel, S | 2011 | 34 | 0.008 | 0.08 | 17 | 0 | 0.116 |
|  | Von Stengel, S | 2011 | 29 | 0.002 | 0.088 | 16 | 0 | 0.116 |
|  | Cheng et al. | 2021 | 19 | 0.02 | 0.085 | 10 | -0.01 | 0.079 |
|  | Cheng et al. | 2021 | 18 | 0.03 | 0.089 | 9 | -0.01 | 0.079 |
| Ward’s triangle | Ba＆Cheng | 2016 | 26 | 0.1 | 0.125 | 26 | 0.02 | 0.135 |
|  | Ba＆Cheng | 2017 | 30 | 0.08 | 0.156 | 31 | 0.02 | 0.187 |
|  | Lu | 2016 | 16 | 0.04 | 0.139 | 5 | 0.02 | 0.159 |
|  | Lu | 2016 | 17 | 0.08 | 0.132 | 6 | 0.02 | 0.159 |
|  | Lu | 2016 | 16 | 0.09 | 0.168 | 5 | 0.02 | 0.159 |
|  | Gusi, N | 2006 | 14 | 0.04 | 0.087 | 14 | 0.01 | 0.037 |
|  | Santin-Medeiros, F | 2015 | 19 | -0.01 | 0.101 | 18 | 0 | 0.131 |
|  | Song&Yang | 2021 | 19 | 0.02 | 0.052 | 7 | 0.01 | 0.036 |
|  | Song&Yang | 2021 | 18 | 0.05 | 0.062 | 6 | 0.01 | 0.036 |
|  | Song&Yang | 2021 | 19 | 0.08 | 0.07 | 7 | 0.01 | 0.036 |
|  | Cheng et al. | 2021 | 19 | 0.08 | 0.087 | 10 | 0.02 | 0.069 |
|  | Cheng et al. | 2021 | 18 | 0.1 | 0.087 | 9 | 0.02 | 0.069 |
| Greater trochanter | Ba＆Cheng | 2016 | 26 | 0.06 | 0.115 | 26 | 0 | 0.085 |
|  | Ba＆Cheng | 2017 | 30 | 0.08 | 0.2 | 31 | 0.01 | 0.154 |
|  | Lu | 2016 | 16 | 0.03 | 0.208 | 5 | -0.01 | 0.105 |
|  | Lu | 2016 | 17 | 0.05 | 0.191 | 6 | -0.01 | 0.105 |
|  | Lu | 2016 | 16 | 0.07 | 0.174 | 5 | -0.01 | 0.105 |
|  | Gusi, N | 2006 | 14 | 0.01 | 0.026 | 14 | -0.01 | 0.035 |
|  | Santin-Medeiros, F | 2015 | 19 | -0.01 | 0.09 | 18 | -0.03 | 0.108 |
|  | Song&Yang | 2021 | 19 | 0 | 0.044 | 7 | 0.02 | 0.053 |
|  | Song&Yang | 2021 | 18 | 0.07 | 0.095 | 6 | 0.02 | 0.053 |
|  | Song&Yang | 2021 | 19 | 0.07 | 0.10 | 7 | 0.02 | 0.053 |
|  | Cheng et al. | 2021 | 19 | 0.05 | 0.098 | 10 | 0 | 0.089 |
|  | Cheng et al. | 2021 | 18 | 0.07 | 0.087 | 9 | 0 | 0.089 |
| Lumbar spine L2-L4 | Ba＆Cheng | 2016 | 26 | 0.07 | 0.207 | 26 | 0.02 | 0.154 |
|  | Shen et al. | 2017 | 18 | 0.03 | 0.135 | 18 | -0.03 | 0.145 |
|  | Shen et al. | 2017 | 20 | 0.04 | 0.181 | 20 | -0.04 | 0.079 |
|  | Song&Yang | 2021 | 19 | 0.02 | 0.098 | 7 | 0.01 | 0.096 |
|  | Song&Yang | 2021 | 18 | 0 | 0.095 | 6 | 0.01 | 0.096 |
|  | Song&Yang | 2021 | 19 | 0.07 | 0.106 | 7 | 0.01 | 0.096 |
| Lumbar spine L1-L4 | Lu et al. | 2012 | 38 | 0.062 | 0.069 | 32 | 0.063 | 0.073 |
|  | Gómez-Cabello,A | 2014 | 24 | 0.01 | 0.17 | 25 | 0 | 0.195 |
|  | Von Stengel, S | 2011 | 34 | 0.005 | 0.14 | 17 | -0.004 | 0.162 |
|  | Von Stengel, S | 2011 | 29 | 0.007 | 0.156 | 16 | -0.004 | 0.162 |
|  | Von Stengel, S | 2011 | 46 | 0.01 | 0.155 | 48 | 0.004 | 0.167 |
|  | Von Stengel, S | 2009 | 44 | 0.011 | 0.155 | 47 | 0.003 | 0.168 |
| Total hip bone | Leung, K. S | 2014 | 280 | -0.95 | 2.519 | 316 | -1.04 | 2.721 |
|  | Santin-Medeiros, F | 2015 | 19 | -0.02 | 0.105 | 18 | -0.03 | 0.137 |
|  | Von Stengel, S | 2011 | 46 | 0.01 | 0.129 | 48 | -0.001 | 0.136 |
|  | Von Stengel, S | 2009 | 44 | 0 | 0.125 | 47 | -0.008 | 0.136 |

Note: Red indicates data after splitting.
