# Supplementary material for: Whole-body vibration training and bone mineral density in older adults: an updated systematic review and meta-analysis
Source: BMC Musculoskelet Disord. 2026 Jan 21;27:149. doi: 10.1186/s12891-026-09504-7 (PMC12908257; doi:10.1186/s12891-026-09504-7)
Supplement: Supplementary file 4 — Supplementary Material 4. [file 12891_2026_9504_MOESM4_ESM.docx]

**Table 2 Methodological quality assessment questionnaire**

| study | Research quality | | | | | memoir | | | | | | | | | | total points | class  level |
| --- | --- | --- | --- | --- | --- | --- | --- | --- | --- | --- | --- | --- | --- | --- | --- | --- | --- |
|  | Eligibility | Designated  randomization | Blinding  of  allocation | Baseline similarity groups | Assessors' blinding | Participant  Compliance  >85% | Adverse events | Exercise adherence | Intention-to-treat analysis | Primary outcome | Secondary outcomes | Point estimates of all measured indicators | Activity monitoring in the control group | Relative exercise intensity was maintained | Exercise volume and capacity expenditure |  |  |
| Ba＆Cheng (2016)^[46]^ | 1 | 0 | 0 | 1 | 0 | 0 | 0 | 0 | 1 | 1 | 0 | 1 | 1 | 1 | 1 | 8 | Moderate |
| Ba＆Cheng (2017)^[47]^ | 1 | 0 | 0 | 1 | 0 | 0 | 0 | 0 | 1 | 1 | 0 | 1 | 1 | 1 | 1 | 8 | Moderate |
| Cheng et al. (2021)^[48]^ | 1 | 0 | 0 | 1 | 0 | 0 | 0 | 0 | 0 | 1 | 0 | 1 | 1 | 1 | 1 | 7 | Moderate |
| Gómez-Cabello et al. (2014)^[49]^ | 1 | 0 | 0 | 1 | 0 | 1 | 1 | 0 | 1 | 1 | 1 | 1 | 0 | 1 | 1 | 10 | Good |
| Gusi et al. (2006)^[50]^ | 1 | 0 | 0 | 0 | 0 | 1 | 1 | 1 | 0 | 1 | 1 | 1 | 1 | 1 | 1 | 10 | Good |
| Leung et al. (2014)^[51]^ | 1 | 1 | 1 | 1 | 1 | 0 | 1 | 1 | 1 | 1 | 1 | 1 | 0 | 1 | 1 | 13 | Excellent |
| Lu (2016)^[52]^ | 1 | 0 | 0 | 1 | 0 | 0 | 0 | 0 | 0 | 1 | 1 | 1 | 1 | 1 | 1 | 8 | Good |
| Lu et al. (2012)^[53]^ | 1 | 0 | 0 | 1 | 0 | 1 | 1 | 0 | 1 | 1 | 1 | 1 | 0 | 1 | 1 | 10 | Good |
| Santin-Medeiros et al. (2015)^[54]^ | 1 | 1 | 0 | 0 | 0 | 0 | 0 | 0 | 0 | 1 | 1 | 1 | 0 | 1 | 1 | 7 | Moderate |
| Shen et al. (2017)^[55]^ | 1 | 0 | 0 | 1 | 0 | 0 | 0 | 0 | 1 | 1 | 1 | 1 | 0 | 1 | 1 | 8 | Moderate |
| Song&Yang (2021)^[56]^ | 1 | 0 | 0 | 1 | 0 | 0 | 0 | 0 | 0 | 1 | 0 | 1 | 0 | 1 | 1 | 6 | Moderate |
| Von Stengel et al. (2009)^[57]^ | 1 | 0 | 0 | 1 | 0 | 0 | 0 | 1 | 1 | 1 | 1 | 1 | 0 | 1 | 1 | 9 | Good |
| Von Stengel et al. (2011a)^[58]^ | 1 | 1 | 1 | 1 | 1 | 0 | 1 | 1 | 1 | 1 | 1 | 1 | 1 | 1 | 1 | 14 | Excellent |
| Von Stengel et al. (2011b)^[59]^ | 1 | 1 | 1 | 1 | 1 | 1 | 1 | 1 | 1 | 1 | 1 | 1 | 1 | 1 | 1 | 15 | Excellent |
