# Supplementary material for: Whole-body vibration training and bone mineral density in older adults: an updated systematic review and meta-analysis
Source: BMC Musculoskelet Disord. 2026 Jan 21;27:149. doi: 10.1186/s12891-026-09504-7 (PMC12908257; doi:10.1186/s12891-026-09504-7)
Supplement: Supplementary file 5 — Supplementary Material 5. [file 12891_2026_9504_MOESM5_ESM.docx]

Subgroup Analysis Forest Plot


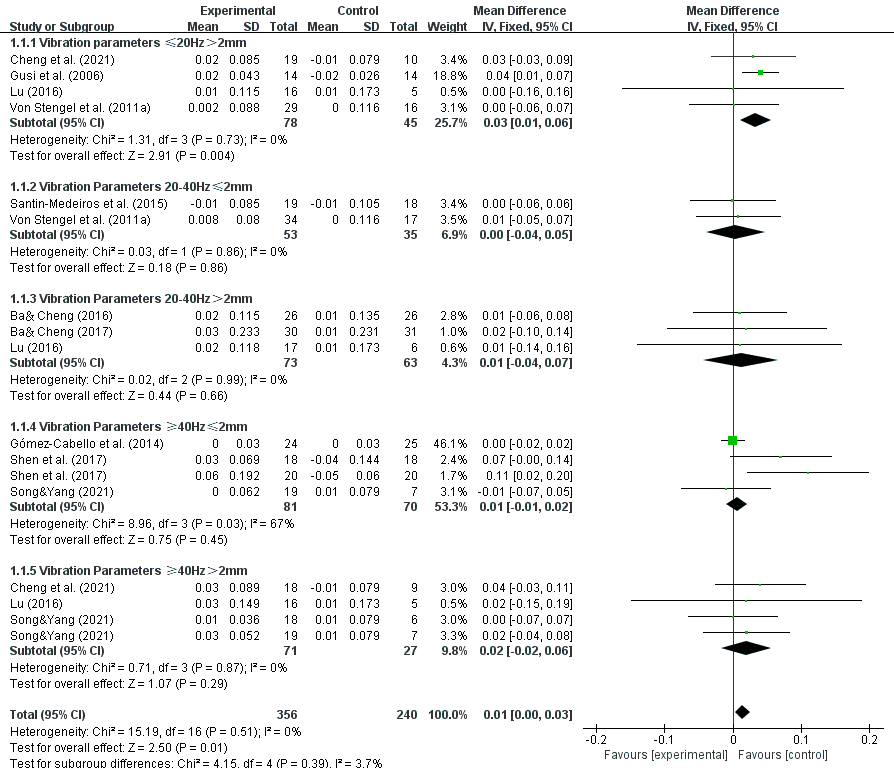


**Figure 1 Forest plot of subgroup analysis of the femoral neck bone mineral density based on vibration parameters**

**
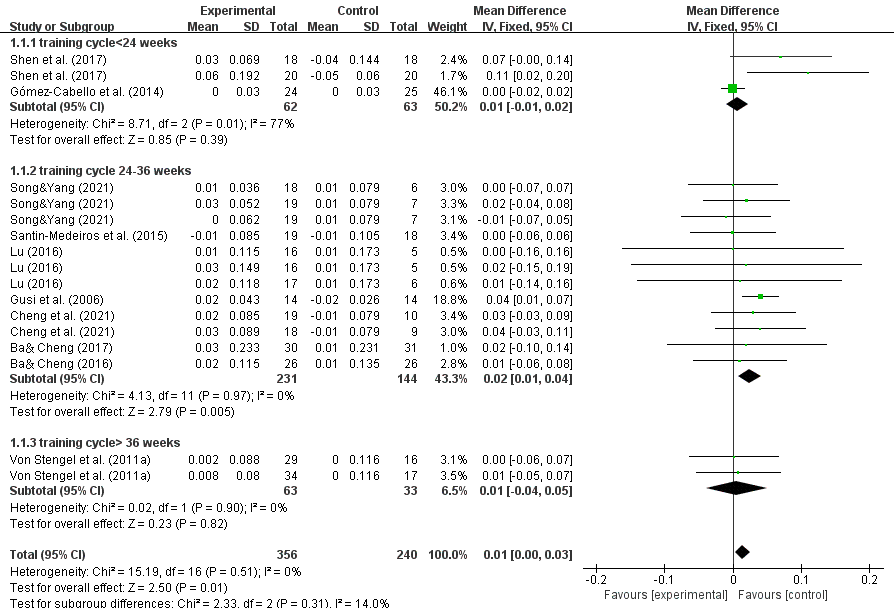
**

**Figure 2 Forest plot of subgroup analysis of the femoral neck bone mineral density based on**

**training cycle**

**
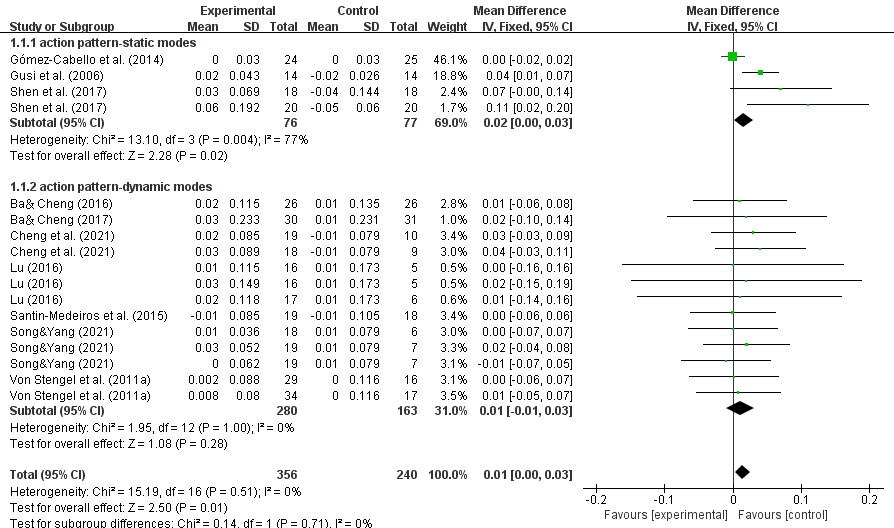
**

**Figure 3 Forest plot of subgroup analysis of the femoral neck bone mineral density based on**

**action pattern**

**
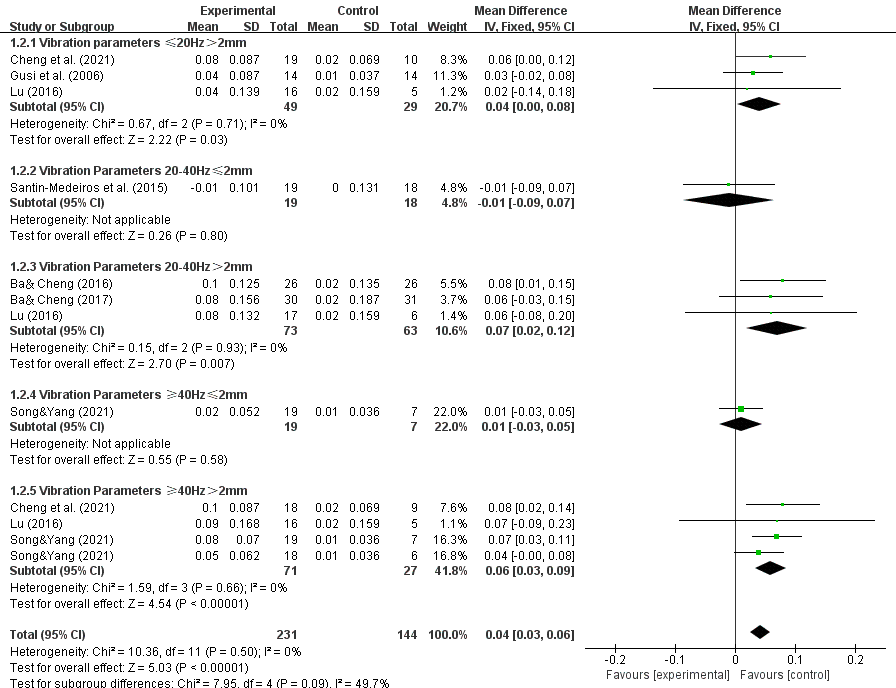
**

**Figure 4 Forest plot of bone density subgroup analysis in the Ward's triangle region based on vibration parameters**

**
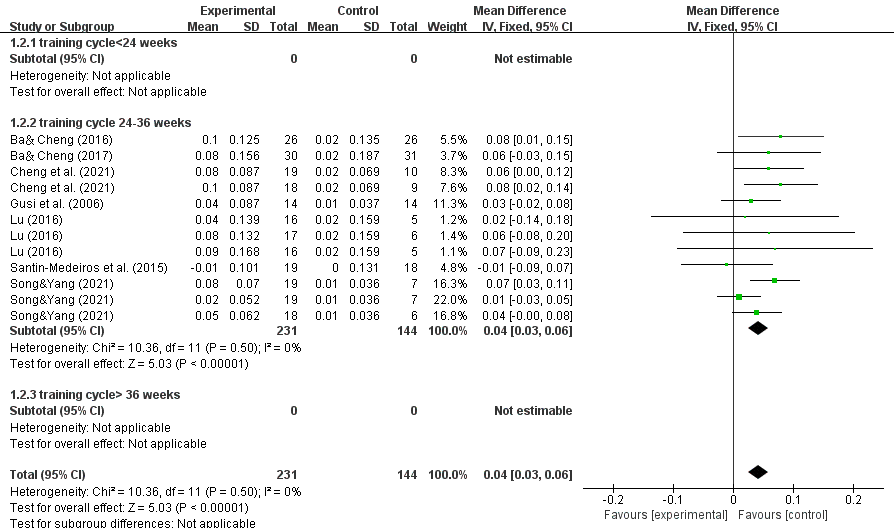
**

**Figure 5 Forest plot of bone density subgroup analysis in the Ward's triangle region based on training cycle**

**
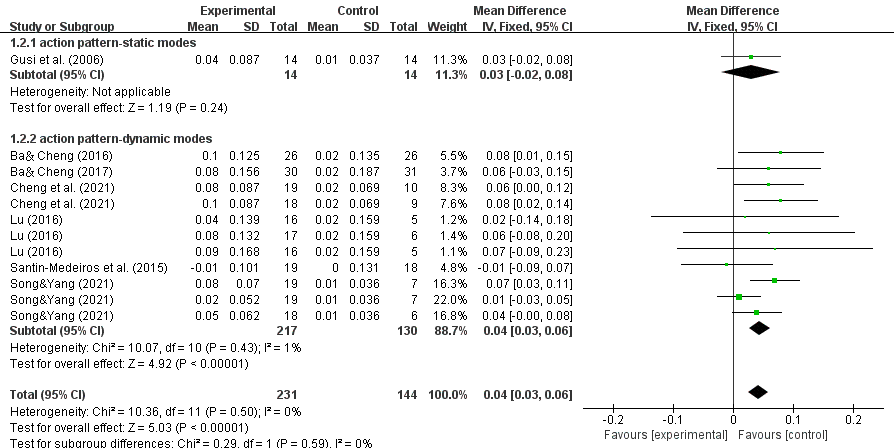
**

**Figure 6 Forest plot of bone density subgroup analysis in the Ward's triangle region based on action pattern**

**
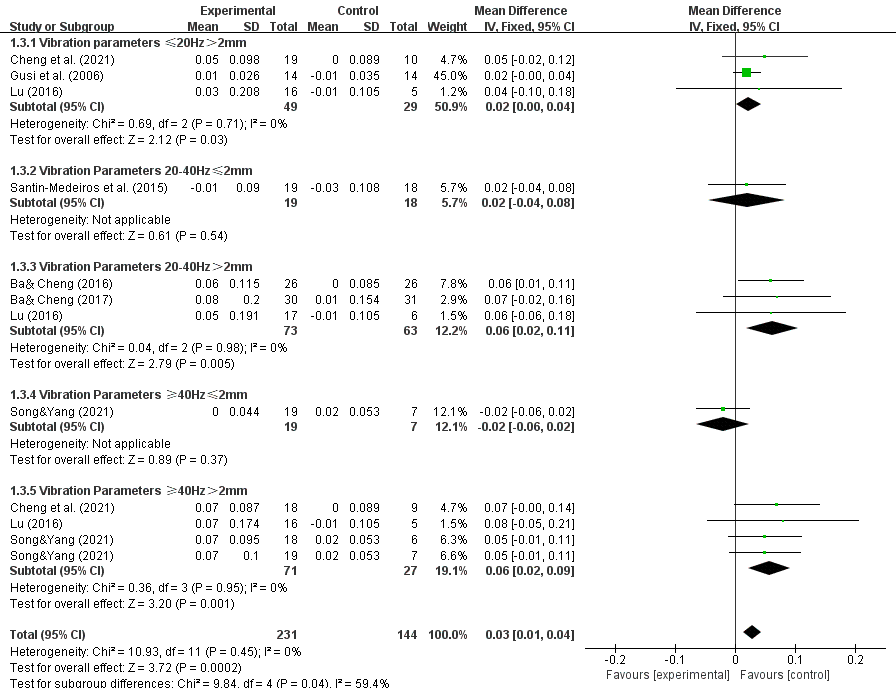
**

**Figure 7 Forest plot of subgroup analysis of the greater trochanter bone mineral density based on vibration parameters**

**
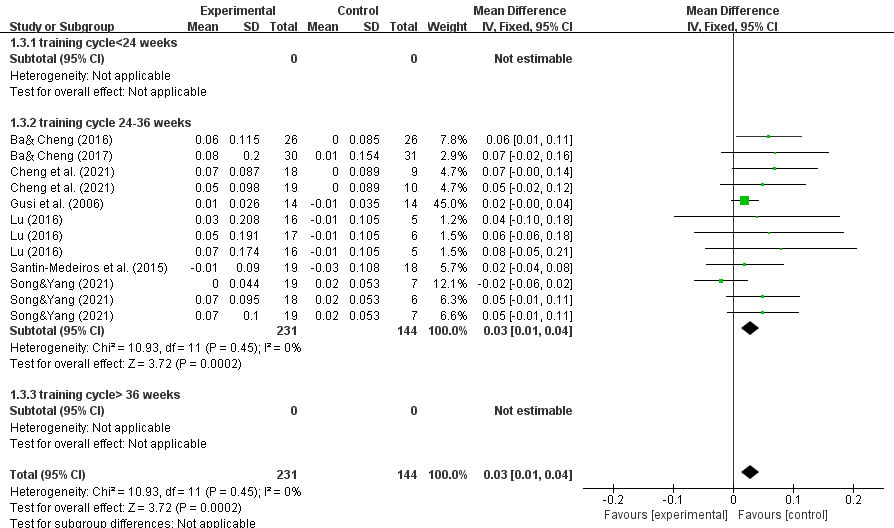
**

**Figure 8 Forest plot of subgroup analysis of the greater trochanter bone mineral density based on training cycle**

**
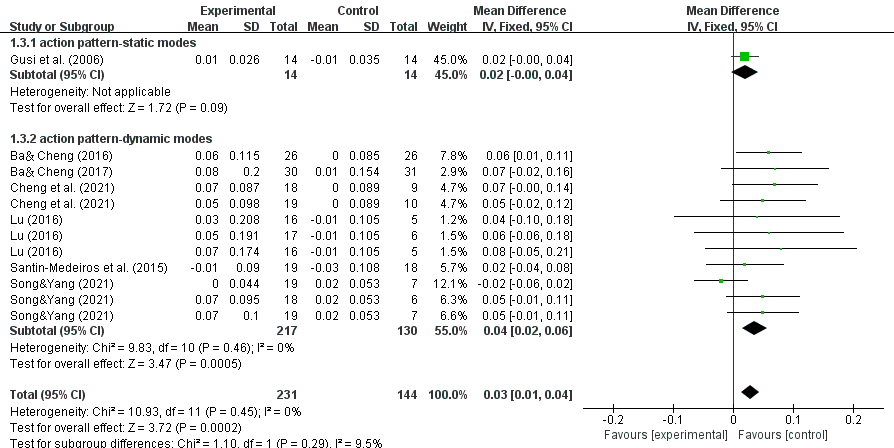
**

**Figure 9 Forest plot of subgroup analysis of the greater trochanter bone mineral density based on action pattern**

**
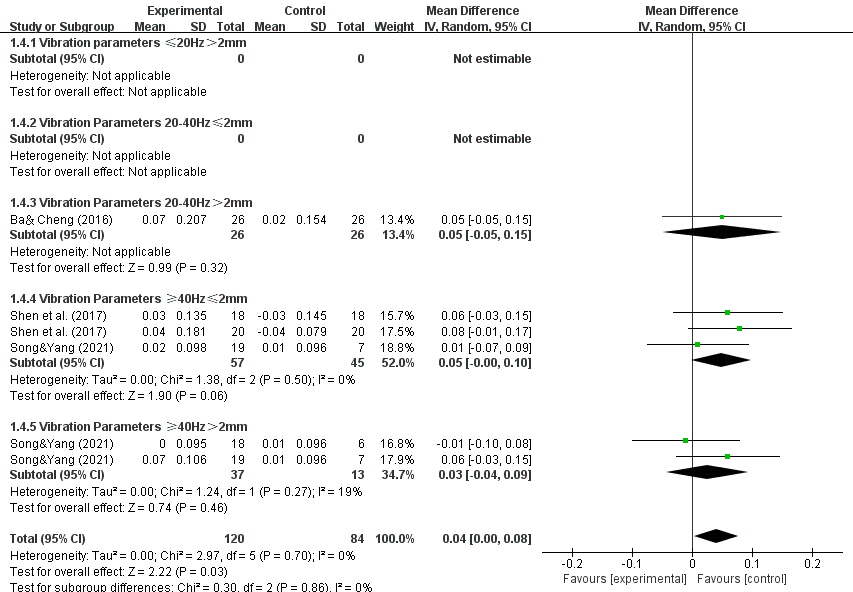
**

**Figure 10 Forest plot of subgroup analysis of the lumbar spine L2‒L4 bone mineral density based on vibration parameters**

**
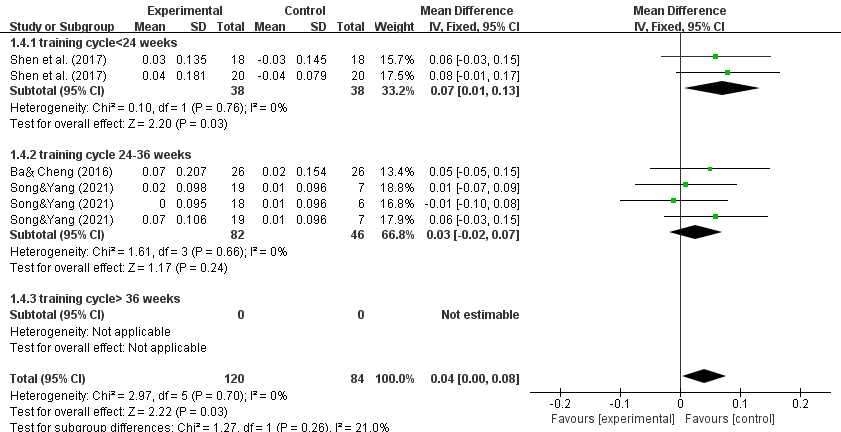
**

**Figure 11 Forest plot of subgroup analysis of the lumbar spine L2‒L4 bone mineral density based on training cycle**

**
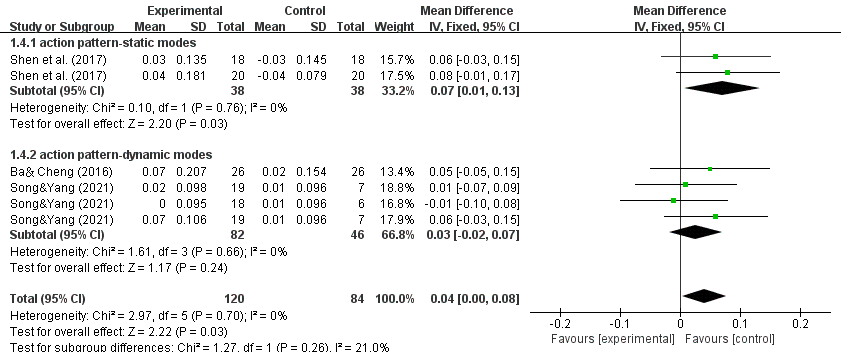
**

**Figure 12 Forest plot of subgroup analysis of the lumbar spine L2‒L4 bone mineral density based on action pattern**

**
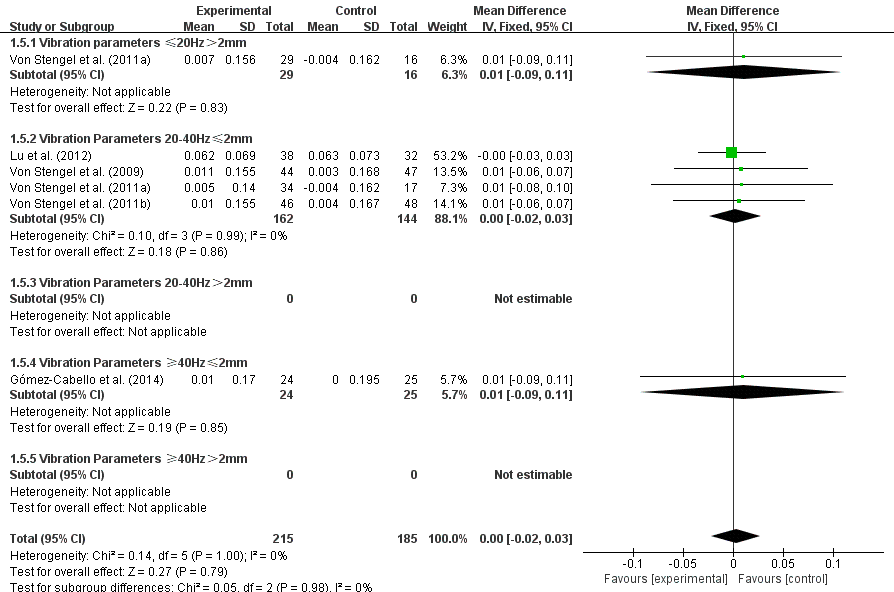
**

**Figure 13 Forest plot of subgroup analysis of the lumbar spine L1‒L4 bone mineral density based on vibration parameters**

**
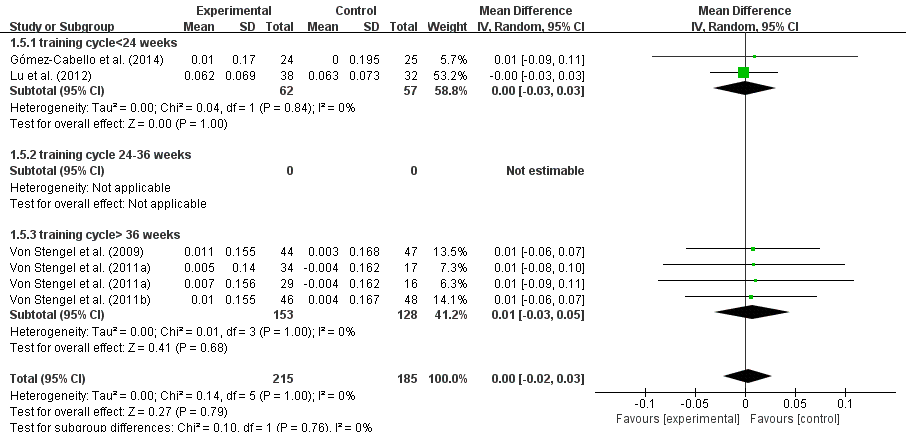
**

**Figure 14 Forest plot of subgroup analysis of the lumbar spine L1‒L4 bone mineral density based on training cycle**

**
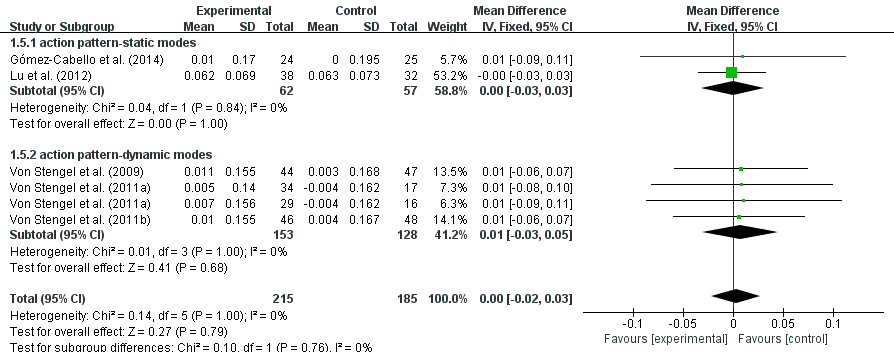
**

**Figure 15 Forest plot of subgroup analysis of the lumbar spine L1‒L4 bone mineral density based on action pattern**

**
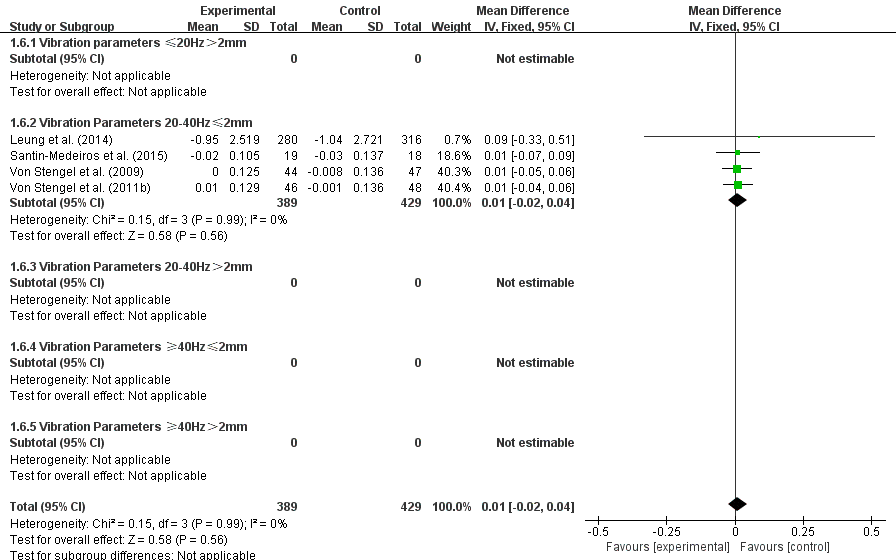
**

**Figure 16 Forest plot of subgroup analysis of the total hip bone bone mineral density based on vibration parameters**

**
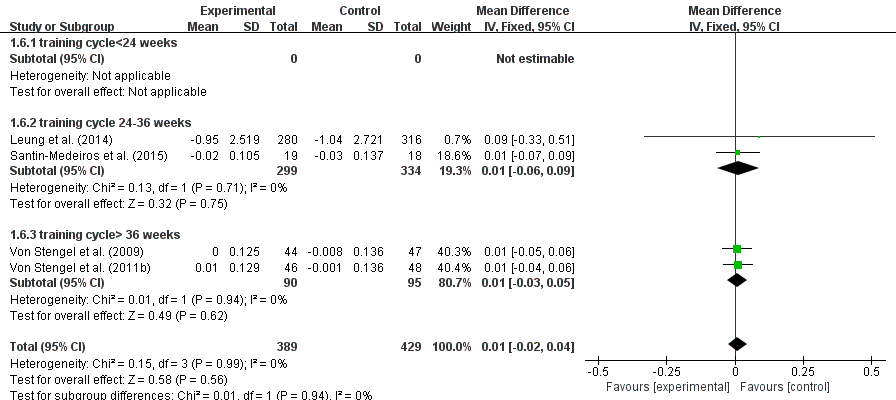
**

**Figure 17 Forest plot of subgroup analysis of the total hip bone bone mineral density based on training cycle**

**
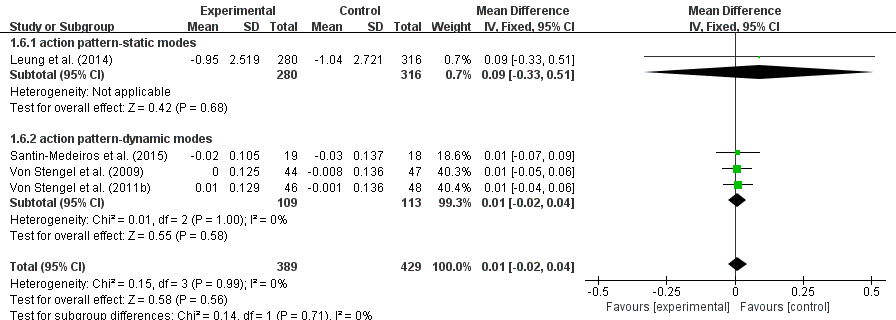
**

**Figure 18 Forest plot of subgroup analysis of the total hip bone bone mineral density based on action pattern**
