# Supplementary material for: Whole-body vibration training and bone mineral density in older adults: an updated systematic review and meta-analysis
Source: BMC Musculoskelet Disord. 2026 Jan 21;27:149. doi: 10.1186/s12891-026-09504-7 (PMC12908257; doi:10.1186/s12891-026-09504-7)
Supplement: Supplementary file 6 — Supplementary Material 6. [file 12891_2026_9504_MOESM6_ESM.docx]

**Supplement 6 sensitivity analysis**

**
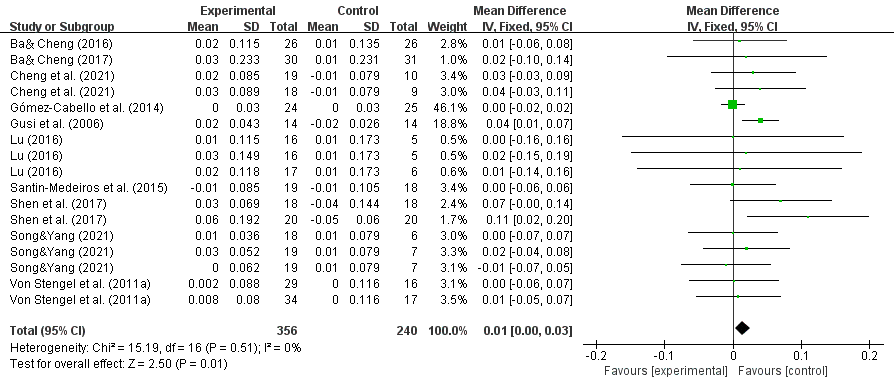
**

**
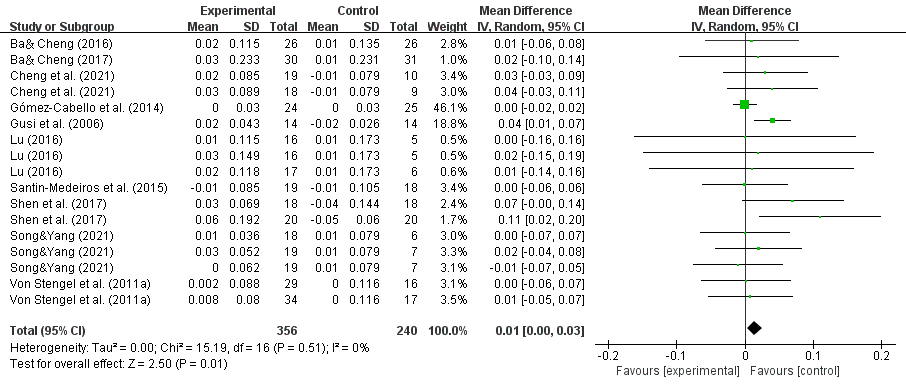
**

**Results of Fixed-Effect and Random-Effect Models on the Effects of Whole-Body Vibration Training on the Femoral Neck Bone Mineral Density Values in Older Adults**

**
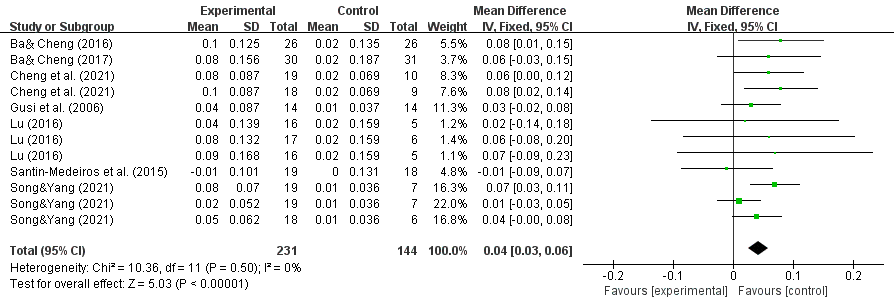

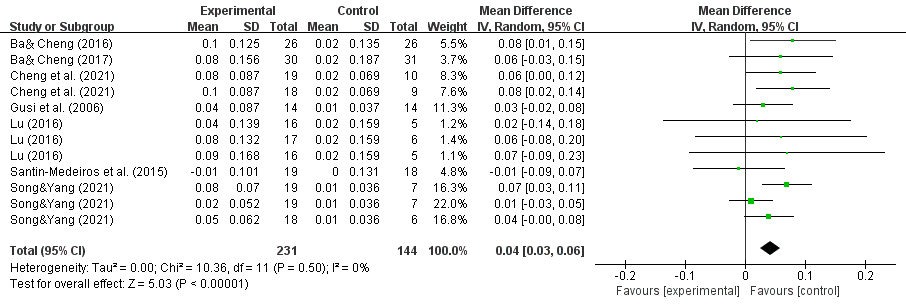
**

**Results of Fixed-Effect and Random-Effect Models on the Effects of Whole-Body Vibration Training on the Ward's triangle Bone Mineral Density Values in Older Adults**

**
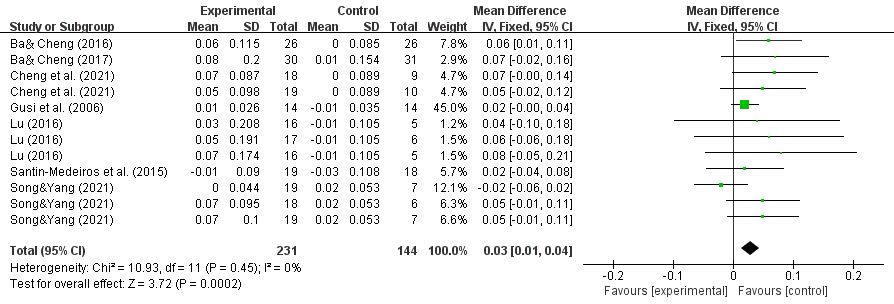

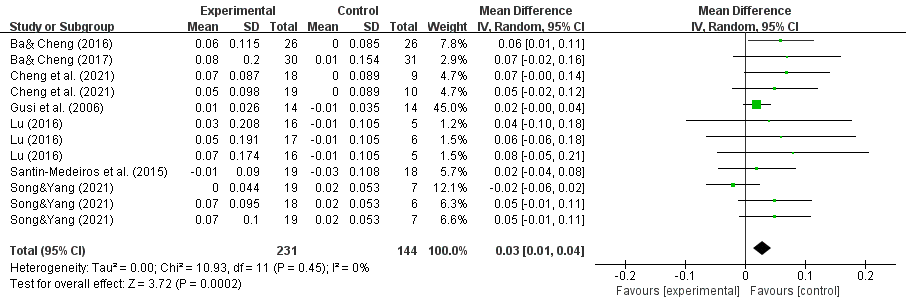
**

**Results of Fixed-Effect and Random-Effect Models on the Effects of Whole-Body Vibration Training on the greater trochanter Bone Mineral Density Values in Older Adults**

**
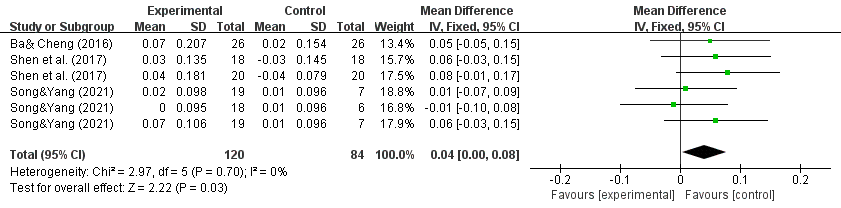

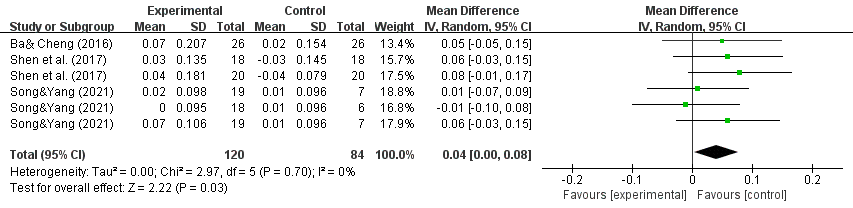
**

**Results of Fixed-Effect and Random-Effect Models on the Effects of Whole-Body Vibration Training on the lumbar spine L2‒L4 Bone Mineral Density Values in Older Adults**

**
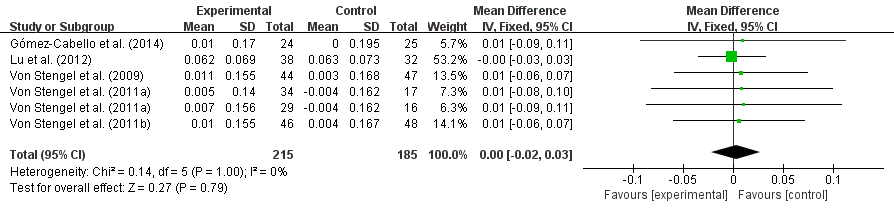

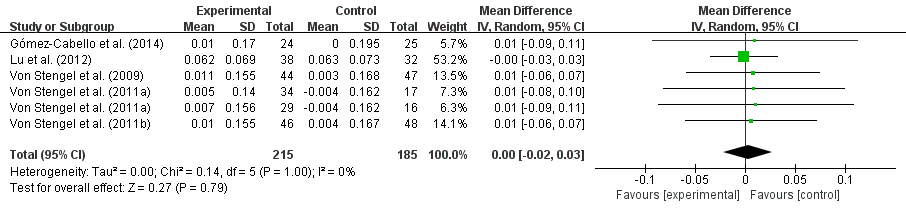
**

**Results of Fixed-Effect and Random-Effect Models on the Effects of Whole-Body Vibration Training on the lumbar spine L1‒L4 Bone Mineral Density Values in Older Adults**

**
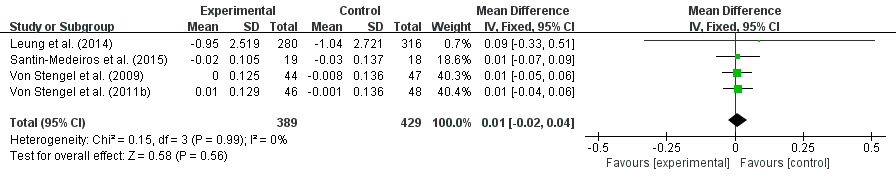

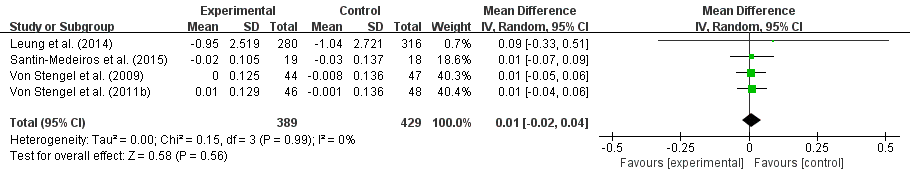
**

**Results of Fixed-Effect and Random-Effect Models on the Effects of Whole-Body Vibration Training on the total hip Bone Mineral Density Values in Older Adults**
